# Enhancing the Solid-State Emission of Carbonyl-Containing Compounds by Means of Introducing a Bifuran Core

**DOI:** 10.1021/acsaom.5c00106

**Published:** 2025-04-21

**Authors:** Hadar R. Yakir, Benny Bogoslavsky, Ori Gidron

**Affiliations:** Institute of Chemistry and the Center for Nanoscience and Nanotechnology, The Hebrew University of Jerusalem, Edmond J. Safra Campus, Jerusalem 9190401, Israel

**Keywords:** oligofurans, oligothiophenes, organic electronic materials, organic light-emitting diodes, fluorescence

## Abstract

Aromatic aldehydes and ketones are attractive as luminescent materials because they exhibit room temperature phosphorescence. However, an absence of significant luminescence in the solid state limits their practical application. This study investigates a series of bifuran dialdehydes and diketones (F1–F3) and their bithiophene analogues (T1–T3) and compares their photophysical properties in solution and the solid state. The incorporation of carbonyl groups into bifuran cores significantly enhances their solid-state fluorescence, with solid-state quantum yields reaching up to 35%, in contrast to low fluorescence for the thiophene-based analogues. Structural analysis via X-ray crystallography reveals that bifuran derivatives exhibit tighter packing and more rigid molecular backbones, which contributes to the observed aggregation-induced emission. The carbonyl group also stabilizes the furan core compared with unmodified bifurans.

## Graphical abstract

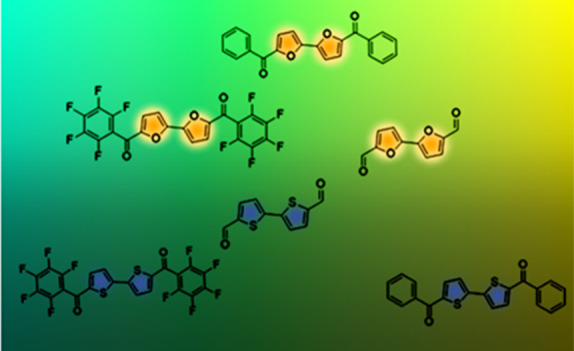

Since the discovery of fluorescein in the mid-19th century, organic luminescent materials have found myriad applications in optoelectronic devices, such as organic light-emitting diodes and sensors.^1,2^ Their unique properties, including tunable emission spectra and ease of processing, have made them attractive candidates for various technologies. Such applications require strong emission in the solid (crystalline, polycrystalline, or amorphous) state.^3,4^ Although many aromatic compounds exhibit fluorescence in solution, most suffer from aggregation-caused quenching (ACQ), which precludes solid state applications.^5^ The discovery of the contrasting phenomena of aggregation-induced emission (AIE) led to a conceptual revolution regarding organic phosphorescence.^6^ Prior to that, the luminescent characteristics of an aggregate were disregarded if there was no emission in solution, and opportunities to study phosphorescence were overlooked. AIE motivates research on aggregates exhibiting room-temperature phosphorescence (RTP).^7^ While several factors can induce AIE, such as the different positioning of luminophores,^8^ the question regarding other factors, such as the replacement of different heteroatoms remains unanswered.

We have previously introduced linear and macrocyclic oligofurans, and found that they confer several advantages over their thiophene analogues, namely stronger fluorescence, better conjugation and delocalization, and tighter packing.^9^ We also found that inclusion of the smallest oligomer fragment, bifuran, is sufficient to obtain many of the favorable properties observed in longer oligomers, such as fluorescence, solubility and planarity.^10^ Despite their advantages, furans suffer from low stability, which limits their applications as luminescent materials in devices. We found that incorporating strong electron withdrawing groups, such as perfluorobenzene, increases their stability significantly.^11^ While Tang observed AIE in tetrafurylethene,^12^ bifuran-containing materials to date did not exhibit AIE.

The inclusion of a carbonyl group often results in contradictory effects. In solution, aromatic aldehydes and ketones can exhibit fast intersystem crossing, which is allowed because mixing occurring between their n−π* and π–π* orbitals enables transition between these states.^13^ In the solid-state, the presence of carbonyl groups often results in quenching of the fluorescence. However, in some crystals the restriction of molecular motions by nonclassical C–H···O hydrogen bonds have been shown to increase luminescence.^14^ In addition, if the packing allows intermolecular electronic coupling between n−π* and π–π*, it can promote RTP.^13^ Some aromatic aldehydes and ketones exhibit AIE whereas others exhibit ACQ, and it is difficult to predict the behavior of a specific compound. Unlike benzene or thiophene aldehydes, 2-furan aldehyde (also known as furfural, from the Latin word for bran) can be obtained directly from distilling agricultural biowaste.^16,17^ However, despite the availability of bifuran dialdehyde, the optical and structural properties of this compound and its derivatives have not been reported.

Combining the features of aromatic ketones and aldehydes with those of a bifuran core, we were interested in exploring bifuran dialdehydes and diketones (F1–F3, Scheme 1), and comparing them with their bithiophene analogues (T1–T3, Scheme 1). Here we report on the structural and photophysical properties of a series of bifuran dialdehydes and diketones in solution and in the solid state. We utilized phenyl ketones and perfluorinated phenyl ketones in light of our previous observations that the perfluorophenyl moiety imparts strong emission to the bifuran core. In addition, we postulated that recently reported strong interactions between the carbonyl groups and the perfluorinated phenyl moiety would result in different properties in the crystalline phase.^18^ We found that, while the X-ray structures of the bifuran compounds resemble those of their bithiophene analogues, they are characterized by shorter intermolecular distances, such that the F1–F3 series exhibits AIE with quantum efficiencies that are up to 10 times higher than their thiophene analogues.

**Scheme 1:**
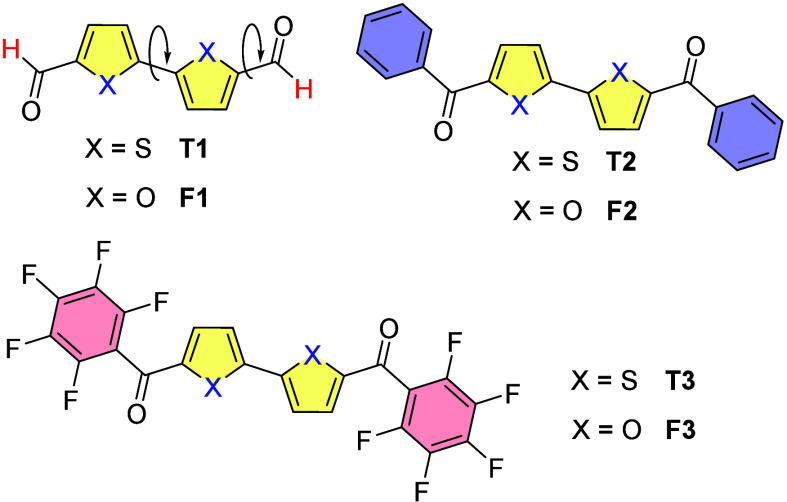
Structural Formulas of Bifurans F1–F3 and Bithiophenes T1–T3

All six candidates were synthesized by oxidative dimerization of the corresponding ketones or aldehydes with palladium acetate (0.15 equiv)^19^ with 36–69% yields (Scheme 2),^20^ and characterized using ^1^H and ^13^C NMR and high-resolution mass spectrometry.

**Scheme 2:**
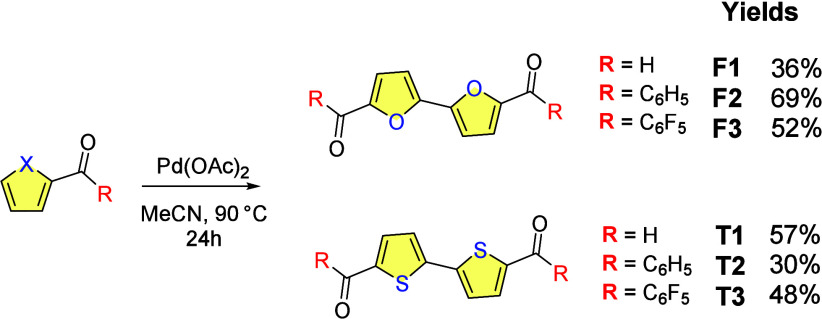
General Synthesis and Yields of Bifurans F1–F3 and Bithiophenes T1–T3

The compounds were crystallized in dichloromethane (DCM) solutions, with their single-crystal X-ray structures depicted in Figure 1 (the structure for T1 was previously reported^21^). While both bifuran and bithiophene cores are planar, the interring bond is shorter for the bifuran cores (1.43 vs 1.45 Å) as is the bond length between the furan/thiophene rings and the carbonyl group (1.44 vs 1.46 Å). This suggests a stronger electron delocalization in the bifuran as previously observed.^10^

**Figure 1:**
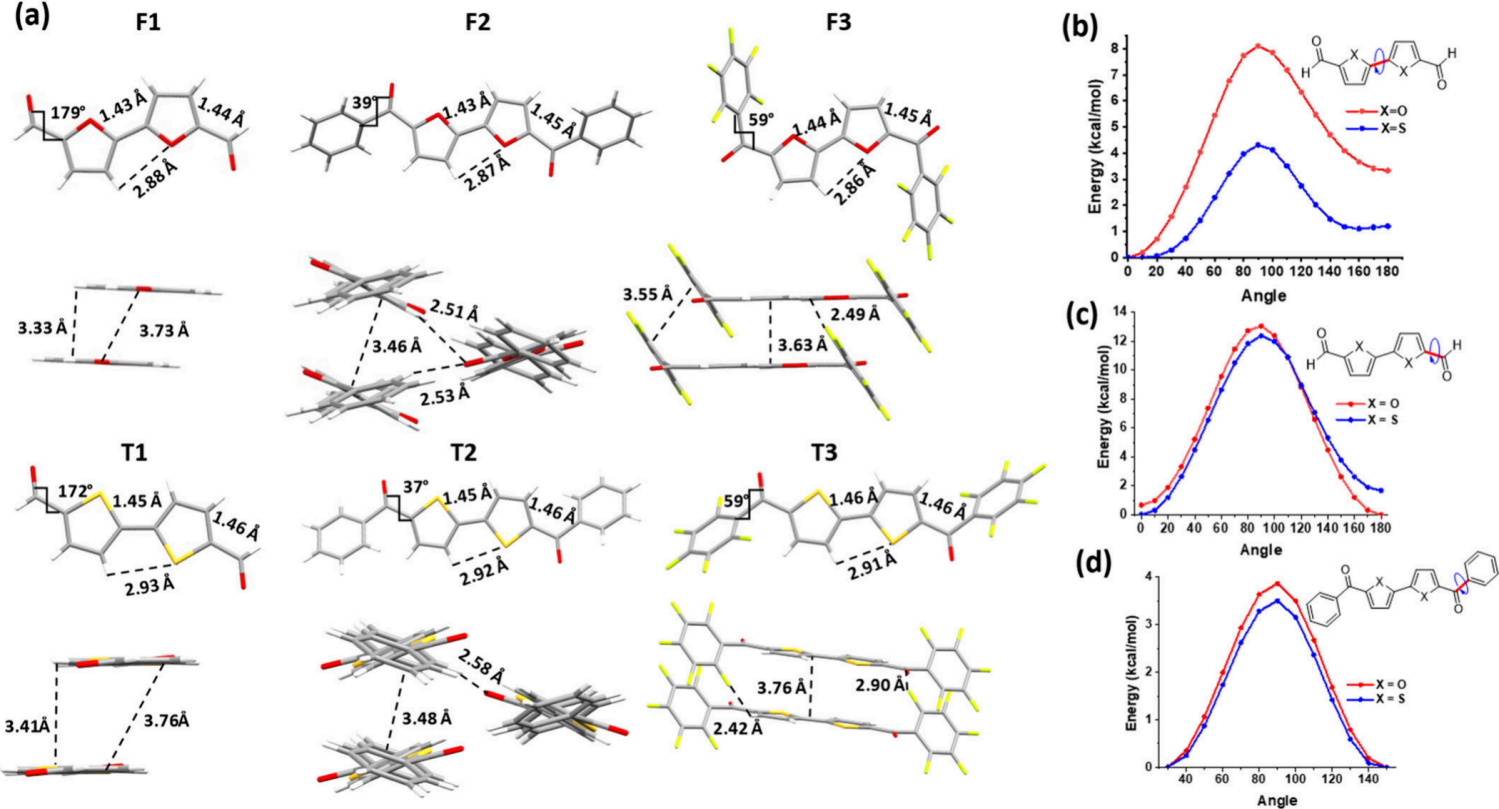
(a) Single-crystal molecular conformations (top) and packing (bottom). (b–d) Calculated rotational barriers for the (b) dimer core in aldehydes, (c) carbonyl moiety in aldehydes, and (d) phenyl moiety in phenyl ketones.

Compounds F1 and F2 show packing motifs similar to those of their thiophene analogues, T1 and T2. However, intermolecular distances are shorter for bifuran derivatives than for bithiophenes; for example, F1 shows a distance of 3.33 Å, compared with 3.41 Å for T1. Similarly, the distances between the carbonyl oxygen and the β-proton are shorter for F2 (2.51 Å) than for T2 (2.58 Å). Thus, overall, the packing is tighter for furan-containing molecules.

One notable feature is the structure of F3, where the carbonyl oxygen is positioned *anti* to the heteroatom, a configuration that significantly alters its packing motif, distinguishing it from its thiophene analogue (Figure 1a). In addition, F3 exhibits a weak C=O···H interaction that contributes to its packing. This interaction stabilizes the polymeric structure formed by F3 molecules, further enhancing the stability of the overall molecular arrangement (Figure S12).

The shorter interring bonds in F*n* are expected to be expressed in a more rigid backbone. This can be observed in a higher barrier of rotation [calculated at DFT/B3LYP/6-311G­(d)/D3] for all interring bonds in F*n* than in T*n*.^23^ The barrier for bifuran rotation is 4 kcal/mol higher than its bithiophene analogue (Figure 1b). The *anti* conformer of the bifuran core is more stable than the *syn* conformer by 4 kcal/mol while a smaller difference of 1.5 kcal is observed for the bithiophene core. The smaller energy difference between the *syn* and *anti* conformers for the bithiophene core is explained by a more significant repulsion of the β-hydrogens with the lone pair of the sulfur atoms. The delocalized nature of the bifuran moiety also affects more distant bonds: the preferred furan–carbonyl orientation is *syn*, while for thiophene, the *anti* orientation is slightly lower in energy (Figure 1d). This can explain the difference observed between the conformation of the crystal structures of F3 and T3. Overall, the delocalized nature of the bifuran core is expressed in more rigid bonds throughout the conjugated backbone.

Unlike diketones and dialdehydes segregated intramolecularly by single aromatic rings, as occurs in the packing of aromatic phenones,^24^ we found that the intermolecular distances between the carbonyl oxygen and the rings of neighboring molecules in the F*n* and T*n* series are too large to afford n−π interactions. This can be rationalized by both stronger π–π interactions in our series, arising from their incorporating a larger π-conjugated moiety than benzene, and by the electron-donating nature of carbonyl groups when they interact with arenes.^25^ The lack of n−π interactions indicates that RTP is not expected in F*n* and T*n* crystals.

In DCM solution, the absorption spectra of F1–F3 display sharper vibronic shoulders than those of T1–T3 (Figure 2, red trace), indicating a more rigid backbone, as mentioned above.^10^ In contrast, the emission spectra of F1–F3 are relatively broad and featureless whereas those of T1–T3 are sharper with clear vibronic shoulders (Figure 2, blue trace). This contrasts with the emission spectra of previously investigated bifuran derivatives, demonstrating sharper peaks compared with those of their thiophene analogues.^10,26^ The emission spectra for F1–F3 also exhibit larger Stokes shifts than those of their thiophene analogues. Thus, the carbonyl moiety significantly modifies the excited state of F1–F3. At room temperature, F2 and F3 emit very weak light in the blue region (quantum efficiency, Φ_f_ < 0.01), and the fluorescence intensity is not significantly affected by the removal of oxygen. In contrast, F1 only exhibits fluorescence under oxygen-free conditions. At 160 K, the emission intensity increases for all compounds, while the shape remains featureless for F1 and T2 even at low temperatures (Figure S17).

**Figure 2:**
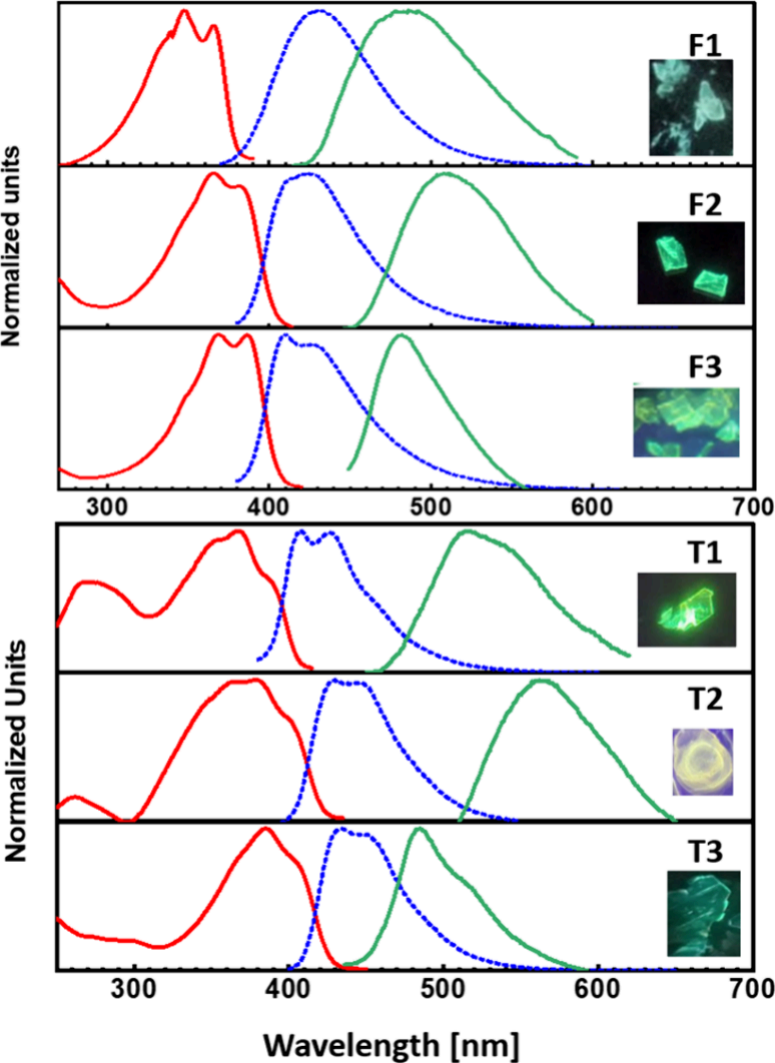
Normalized absorption (red line) and emission spectra in DCM (blue line) and in the solid state (green line). Excitation wavelength: in DCM, F1–F3 and T3 at 365 nm and T2 and T3 at 380 nm, and in the solid state, F1–F3 and T1 at 415 nm, T2 at 430 nm, and T3 at 370 nm. All measured in ambient conditions, except F1, which was measured under N_2_.

In the solid state (Figure 2, green trace), the emission spectra are significantly bathochromically shifted for all compounds. The most significant difference is observed for F2, for which the solid-state emission is bathochromically shifted by 0.50 eV compared with its emission in solution. Upon cooling to 100 K, the solid-state emission is further red-shifted and more intense for all compounds.

The fluorescence quantum efficiency in solution is very low for all compounds, and especially for F1–F3 (Φ_f_ < 0.01), whereas it is slightly higher (Φ_f_ ≈ 0.05) for T2 and T3. However, for the F1–F3 series, quantum yields are significantly greater in the solid state than in solution (e.g., Φ_f_ < 0.01 for F2 in DCM solution vs Φ_f_ = 0.35 for F2 in the solid state; Table 1 and Figure 3). This is in sharp contrast to their thiophene analogues, which show similar or even weaker emission as solids than as solutions. Thus, bifuran dialdehyde and diketones exhibit AIE whereas their bithiophene analogues exhibit either ACQ or no significant change upon aggregation. The AIE of F1–F3 is clearly demonstrated by comparing their emission intensities in THF solutions versus THF:water mixtures. As the water content increases, there is a noticeable enhancement in emission intensity. (Figure S18). Thus, overall, aggregation induces a strong increase in emission for F1–F3, while for T1–T3 aggregation induces either small increase or quenching of the emission. The CIE coordinates are in the green-cyan range for all compounds except of T2 which is light orange (Figure S15).

**Figure 3:**
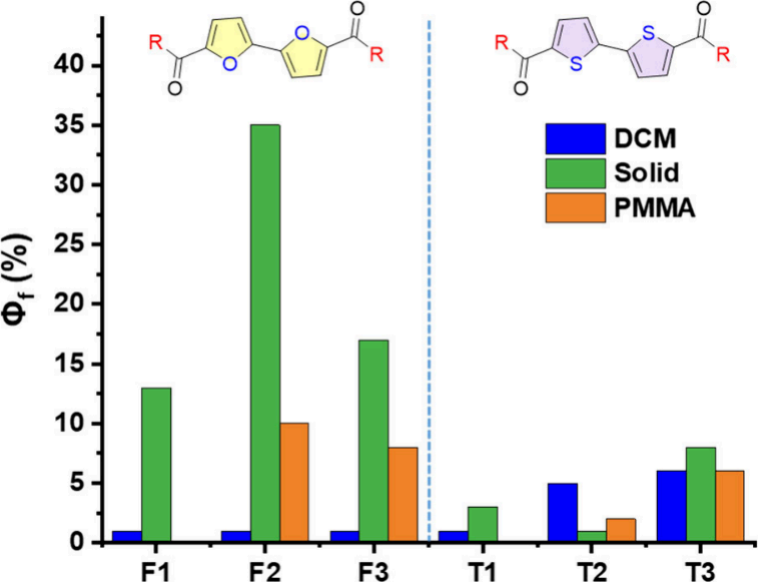
Fluorescence quantum yields (Φ_f_) for biofuran-based (F1–F3) versus biothiophine-based (T1–T3) carbonyl-containing compounds in solution (blue), in the solid state (green), and embedded in PMMA (orange).

**Table 1:** Summary of the Photophysical Properties of the F1–F3 and T1–T3 Series in Solution and the Solid State

	λ_abs_ [nm]	λ_f_ [nm]			Φ_f_ [%]			τ_f_ [ns]	
	solution^a^	solution	solid	PMMA^c^	solution	solid	PMMA	solution	solid
F1	347	436^b^	488		<1	13		1.6	7
F2	365	423	509	424	<1	35	10	0.7	7
F3	369	413	480	450, 470	<1	17	8	0.5	5
T1	368	408	500		<1	3		0.8	5
T2	375	428	560	440	5	2	2	3	0.1
T3	385	430	490, 515	430	6	7	6	0.1	1

a Measured in DCM.

b Measured in an oxygen-free environment.

c No emission was detected from F1 and T1 in PMMA. Variables: fluorescence absorption maximum, λ_abs_; fluorescence emission maximum, λ_f_; fluorescence quantum yield, Φ_f_; fluorescence lifetime, τ_f_; poly­(methyl methacrylate), PMMA. The absorption coefficient (ε) and radiative (*K*_r_) and nonradiative (*K*_nr_) rate constants can be found in Table S3.

To further understand the photophysical differences between furan- and thiophene-based carbonyl-containing compounds, we calculated thiophene and furan dialdehydes and diketones using the DFT-B3LYP/6-311G­(d)/D3 level of theory. The addition of carbonyl groups significantly reduces the HOMO energy level of the bifuran core, bringing it close to that of its bithiophene analogue, with only a 0.1 eV difference (compared with a 0.3 eV difference between parent bifuran/bithiophene). The HOMO–LUMO gap is smaller for the bithiophene analogues by 0.1–0.3 eV. To account for the differences in absorption and emission in the solid (crystalline) state, we calculated the dimer model based on the crystal data, taking the nearest neighbors (Figure S10 and Table S2). Overall, the orbital coupling is more significant for F1–F3 than for T1–T3, despite the smaller heteroatom size in furans than in thiophenes, which can be rationalized by the shorter intermolecular distances.

Several factors can account for the strong AIE in bifuran diketones and dialdehydes compared with their bithiophenes analogues. (1) Tighter crystal packing restricts molecular motions, which results in a competing nonradiative decay (internal conversion) process. (2) The inherent delocalized nature of the bifuran core influences the carbonyl and phenyl interring bond distances, which results in a more rigid backbone, thus minimizing nonradiative relaxation pathways. To differentiate between intermolecular interactions and intramolecular restrictions, we embedded the furan-based chromophores in poly­(methyl methacrylate) (PMMA) films (Figure 3, orange). We found that, whereas the quantum efficiencies of F2 and F3 increase when they are embedded in PMMA compared with solution, they are still lower than those in the crystalline phase. Thus, it seems that the observed AIE results from a combination of molecular movement restriction and intermolecular interactions. In contrast, the quantum efficiencies of the thiophene analogues T2 and T3 are similar under all three conditions (embedded in PMMA, in the crystalline phase, and in DCM solution), which indicates that neither intermolecular nor intramolecular factors diminish the nonradiative decay pathways. Neither of the aldehydes (F1 and T1) showed emission in PMMA.

For F2, T2, and T3, the emission wavelength remains consistent with that in solution, indicating that the observed red shift in the solid state is due to crystal lattice effects (Table 1). By contrast, F3 exhibits a red shift of 20 nm, resulting in an emission wavelength that is intermediate between those observed in solution and in the solid state. This suggests that the red shift for F3 in the solid state arises from both crystal lattice effects and limitations on molecular movement (Figure S19).

Although crystal packing shows almost the same motifs for both F1 and F2 compared with T1 and T2, a closer look reveals a significant difference, with the intermolecular C–H···O distances being much shorter in the furan-containing materials. Figure 4 displays the X-ray structures for F1–F2 and T1–T2 trimers, where the hydrogen atoms were optimized at the CAM-B3LYP/6-31-G­(d)/D3 level while freezing the carbon, oxygen, and sulfur atoms. For both F1 and F2, the distance between the carbonyl oxygen and the nearest hydrogen is at least 0.1 Å shorter than that for the thiophene analogues. Despite having an equal number of close interactions, the F*n* compounds consistently exhibit a shorter distance, albeit with a slight difference. Such nonclassical C–H···O hydrogen bonds “lock” the carbonyl groups in place and thus reduce their degrees of freedom.^27^ Such locking was previously used to explain the emission observed from nonaromatic amino-acids, as well as the phosphorescence observed in aromatic ketones.^27^ In the current case, the smaller size of the furan oxygen than the thiophene sulfur can account for the tighter packing and shorter C–H···O distances in F1–F2 than in T1–T2. In the case of F3 and T3, the comparison is more complex, as the carbonyl oxygen in F3 is positioned *anti* to the furan heteroatom. Consequently, we conclude that the effect of the carbonyl group is less significant in the presence of a perfluorobenzene moiety. Nevertheless, even in these compounds, the carbonyl group in F3 maintains a shorter distance to the neighboring hydrogens than the distance observed in T3 (Figure S11).

**Figure 4:**
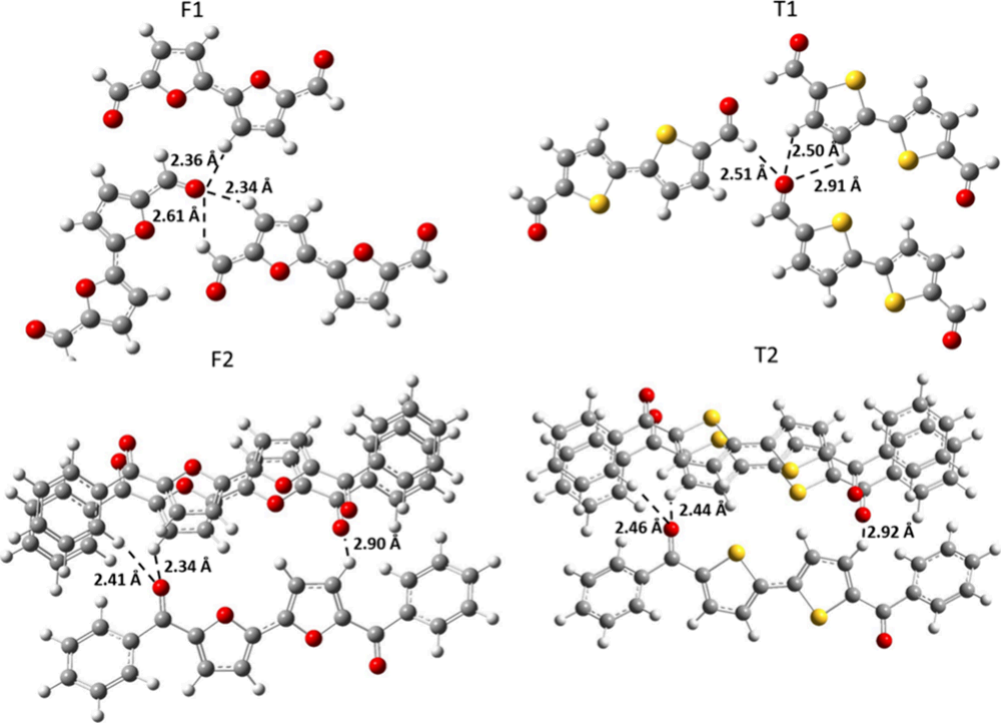
X-ray structures of trimers of F*n* (left) and T*n* (right). For accuracy, the hydrogen atoms were optimized at the CAM-B3LYP/6-31-G­(d)/D3 level.

To further explore the intermolecular interactions in the compounds, we performed a Hirshfeld surface analysis using the crystallographic data (Figure S13), calculated with *CrystalExplorer* software.^29^ The surface is color-coded based on the proximity of atoms within the molecule to its neighbors. Red regions highlight short contacts, indicating areas with strong intermolecular interactions, while blue regions represent longer distances, suggesting weaker interactions or noninteracting areas. In the Hirshfeld surfaces of the F1 and F2 compounds, the red regions around the carbonyl oxygen atoms point to significant intermolecular interactions with hydrogen atoms, which play a crucial role in stabilizing the molecular packing within the crystal. These red areas are more intense than those of T1 and T2, as expected. In the cases of F3 and T3, although red regions are still visible around the carbonyl oxygens, we also observe close interactions near the perfluorobenzene moiety, indicating additional intermolecular interactions.

Although F3 demonstrates tight packing, its Φ_f_ is lower than that of F2. We note that, compared with other F*n* and T*n* molecules, for which the angle between the two electric transition dipole moments in the dipole moment is 80–90°, the angle in F3 is oriented at 54°, which is nearly the magic angle (Figure S22).

We aimed to investigate the impact of incorporating carbonyl group on the stability of the bifuran core by measuring absorption spectra in 1,4-dioxane solution under ambient conditions. Our results indicate that bithiophenes exhibit greater stability than bifurans, with the T2 derivative being the most stable. To further assess the effect of the carbonyl group on stability, we compared these compounds with the previously published 5,5′-diphenyl-2,2′-bifuran,^30^ which decomposes more rapidly than F2. This comparison demonstrates that the presence of the carbonyl group enhances the stability of furan-containing materials (Figure S16).

In conclusion, we introduced a series of aromatic dialdehydes and diketones consisting of bifuran and bithiophene cores. Their X-ray structures display similar packing motifs with shorter distances for the bifuran analogues, and an absence of carbonyl–aryl π interactions. This is likely to result from the presence of stronger π–π interactions in longer conjugated cores. In a consistent manner, F1–F3 display AIE with solid-state quantum efficiencies of up to 35% for F2, whereas their thiophene analogues display either ACQ or are only slightly affected by aggregation, with significantly lower fluorescence. The stronger fluorescence in bifurans is explained by the restriction of internal-conversion pathways, in most cases resulting from shorter CO---H distances in F1–F3. The electron withdrawing carbonyl group significantly stabilizes furan cores, which, combined with their strong luminescence in the solid state, makes bifuran-carbonyl-based materials promising candidates for luminescent devices.

## Supplementary Material













## Data Availability

CCDC 2418472–2418476 contain the supplementary crystallographic data for this paper. These data can be obtained free of charge via www.ccdc.cam.ac.uk/data_request/cif, or by emailing data_request@ccdc.cam.ac.uk, or by contacting The Cambridge Crystallographic Data Centre, 12 Union Road, Cambridge CB2 1EZ, UK; fax: + 44 1223 336033.

## Notes

Experimental procedures, spectral data, X-ray structural data, and computational details (PDF)
Crystallographic data for CCDC 2418472 (CIF)
Crystallographic data for CCDC 2418473 (CIF)
Crystallographic data for CCDC 2418474 (CIF)
Crystallographic data for CCDC 2418475 (CIF)
Crystallographic data for CCDC 2418476 (CIF)
