# Supplementary material for: Enhancing the Solid-State Emission of Carbonyl-Containing Compounds by Means of Introducing a Bifuran Core
Source: ACS Appl Opt Mater. 2025 Apr 21;3(5):1044–9. doi: 10.1021/acsaom.5c00106 (PMC12105016; doi:10.1021/acsaom.5c00106)
Supplement: Supplementary file 1 [file ot5c00106_si_001.pdf]

Supporting Information

Enhancing the Solid-State Emission of Carbonyl-Containing Compounds by Means of Introducing a Bifuran Core

Hadar R. Yakir; Benny Bogoslavsky; and Ori Gidron*

Prof. Ori Gidron, Institute of Chemistry and the Center for Nanoscience and Nanotechnology, The Hebrew University of Jerusalem, Edmond J. Safra Campus, Jerusalem 9190401, Israel

Email: ori.gidron@mail.huji.ac.il

Table of Contents

S1. General 3

S2. General procedure for the synthesis of F2, F3, T2, T3 3

S4. Calculations 9

Excitation energies and oscillator strengths: 10

Cartesian coordinates calculated at B3LYP/6-31G(d) 11

Calculated Dimers 17

S5. X-ray structures of trimers 18

S6. Hirshfeld Surface Analysis 19

S7. Photophysical properties 19

S8. Excitation spectra 20

S9. CIE 1931 coordinate diagram 20

S10. Stability under ambient conditions 21

S11. Photoluminescence at low temperature in solution and solid state 21

S12. PL in THF/water solutions 22

S13. PL in PMMA 23

S14. Measurements in different solvents 23

S15. Lifetime Measurements 24

S16. J-aggregation calculations 25

S17. X-Ray Data Collection and Structure Refinement 26

S18. References 31

S1. General

^{1}H and ^{13}C NMR spectra were recorded in solution on Brücker-AVIII 400 MHz and 500 MHz spectrometer using tetramethylsilane (TMS) as the external standard. Using chloroform-d as the solvent. Chemical shifts are expressed in δ units. Flash chromatography was performed using CombiFlash SiO_2 columns. T1 and F1 were synthesized according to previous reports.[1,2] All calculations were carried out with the Gaussian 09 series of programs[3] using density function theory (DFT). Becke's three parameter exchange functional combined with the Lee−Yang−Parr correlation functional (B3LYP) and with the 6-31G(d) basis set was used for all calculations.[4–7] UV/Vis absorption spectra were recorded on an *Agilent Technologies Cary 5000* UV/Vis-NIR spectrophotometer. Fluorescence measurements were carried out with a Horiba Scientific Fluoromax-4 spectrofluorometer. Absolute fluorescence quantum yield measurements were performed using Quanta-Phi integrating sphere connected to Fluoromax-4. Fluorescence lifetime measurements were obtained using a Fluoro-Hub-B, equipped with NanoLed-*390* as light source. Polymethyl Methacrylate (PMMA; average Mw ∼ 120,000; Sigma Aldrich) was used without any further purification . PMMA films containing a fluorescent compound were prepared by dissolving PMMA in THF (tetrahydrofuran) and incorporating the fluorescent compound into the solution. After thorough mixing, the solution was cast onto a surface and allowed to dry in open air. For single crystal structure determination, a suitable crystal was selected and placed on a XtaLAB synergy, single source at offset/far, HyPix diffractometer. The crystal was kept at 293.69(10) K during data collection. Using Olex2[8], the structure was solved with the ShelXT[9] structure solution program using Intrinsic Phasing and refined with the ShelXL[10] refinement package using Least Squares minimisation.

S2. General procedure for the synthesis of F2, F3, T2, T3

A mixture of monomer (100 mg) and Palladium(II) acetate (15%**)** in dry acetonitrile (20 mL) was stirred for 24 h at 90 °C under argon. The reaction was quenched with water, and the resulting mixture extracted with ethyl acetate (3×10 mL). After removal of the solvent under vacuum, the crude material was purified by flash column chromatography using hexane and ethyl acetate (9:1) over SiO_2**.**

(2,2'-bifuran)-5,5'-diylbis(phenylmethanone) (F2)

69% yield. ^{1}H-NMR (400 MHz, $CDCl_3$): 8.03 – 7.96 (m, 4H), 7.68 – 7.59 (m, 2H), 7.57 – 7.49 (m, 4H), 7.33 (d, J = 3.7 Hz, 2H), 7.06 (d, J = 3.7 Hz, 2H). ^{13}C-NMR (126 MHz, $CDCl_3$): 182.22, 152.27, 148.92, 137.32, 132.94, 129.21, 128.70, 122.53, 110.55.

HRMS (ESI) m/z: [M+H]+ Calcd. 343.0965; found 343.0963.

(2,2'-bifuran)-5,5'-diylbis((perfluorophenyl)methanone) (F3)

52% yield. ^{1}H-NMR (400 MHz, $CDCl_3$): 7.33 (*d*, J=3.9 Hz, 2 H), 7.05 (*d*, *J*=3.9 Hz, 2 H). ^{19}F-NMR (470 MHz, $CDCl_3$): -139.99 (*m,* 2 F); -149.93 (*tt*, 1 F); -160.00 (*m*, 2F).
HRMS (ESI) m/z: [M+H]+ Calcd. 523.0022; found 523.0021.

(2,2'-bithiophene)-5,5'-diylbis(phenylmethanone) (T2)

30% yield. ^{1}H NMR (400 MHz, $CDCl_3$) δ 7.88 (d, *J* = 8.0 Hz, 4H), 7.64 – 7.57 (m, 4H), 7.53 (d, *J* = 7.9 Hz, 4H), 7.38 (d, *J* = 3.9 Hz, 2H)). ^{13}C NMR (126 MHz, CDCl3) δ 187.88, 144.48, 143.73, 137.85, 135.72, 132.67, 129.26, 128.71, 126.14.
HRMS (ESI) m/z: [M+H]+ Calcd. 375.05080; found 375.05168

(2,2'-bithiophene)-5,5'-diylbis((perfluorophenyl)methanone) (T3)

48% yield. ^{1}H NMR (400 MHz, $CDCl_3$) δ 7.47 (dt, *J* = 4.2, 1.2 Hz, 2H), 7.41 (d, *J* = 4.1 Hz, 2H). ^{19}F NMR (376 MHz, $CDCl_3$) δ -140.56 – -140.73 (m), -156.43 (td, *J* = 21.2, 2.6 Hz), -161.95 – -162.22 (m). ^{13}C NMR (126 MHz, $CDCl_3$) δ 146.44, 143.31, 136.70, 127.24.
HRMS (ESI) m/z: [M+H]+ Calcd. 552.94203; found 552.94275.

S3. NMR spectra

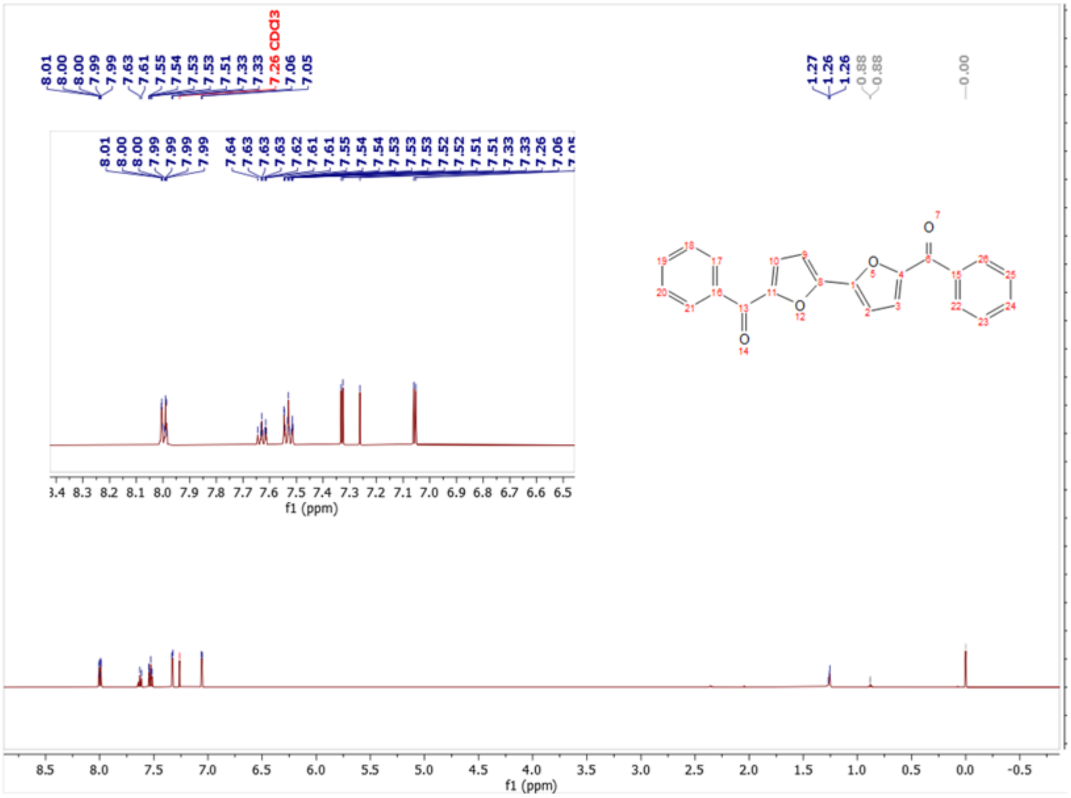

Figure S1. **^{1}H-NMR** spectrum of **F2** in chloroform-*d*

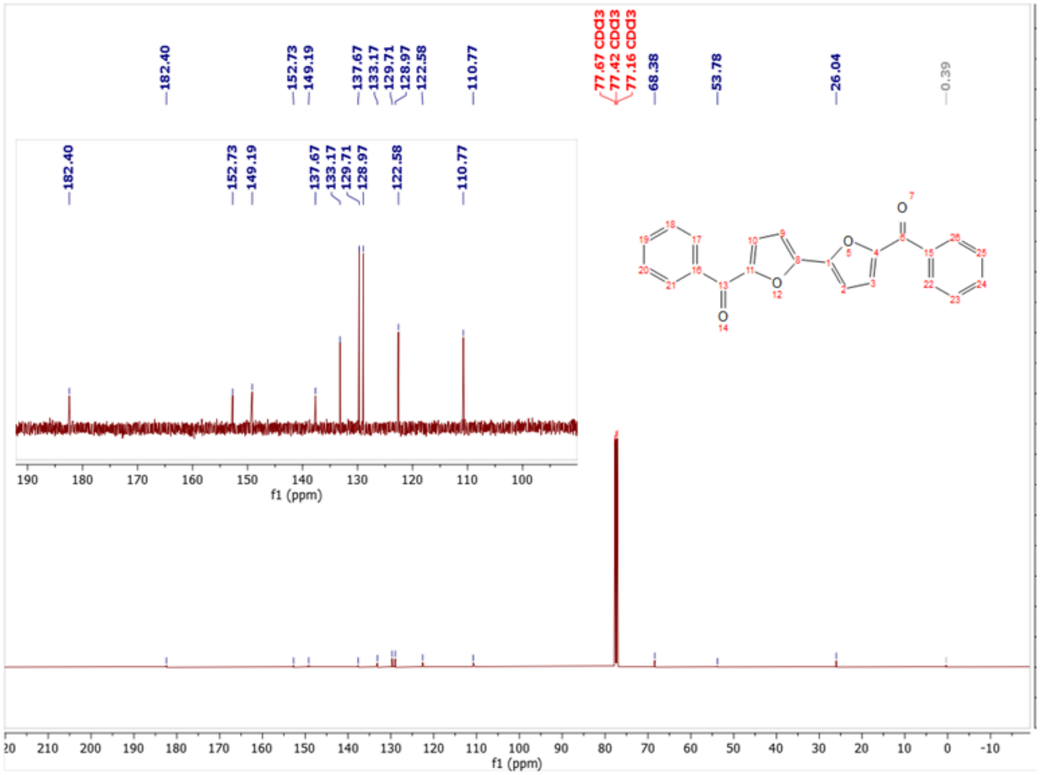

Figure S2. **^{13}C-NMR** spectrum of **F2** in chloroform-*d*

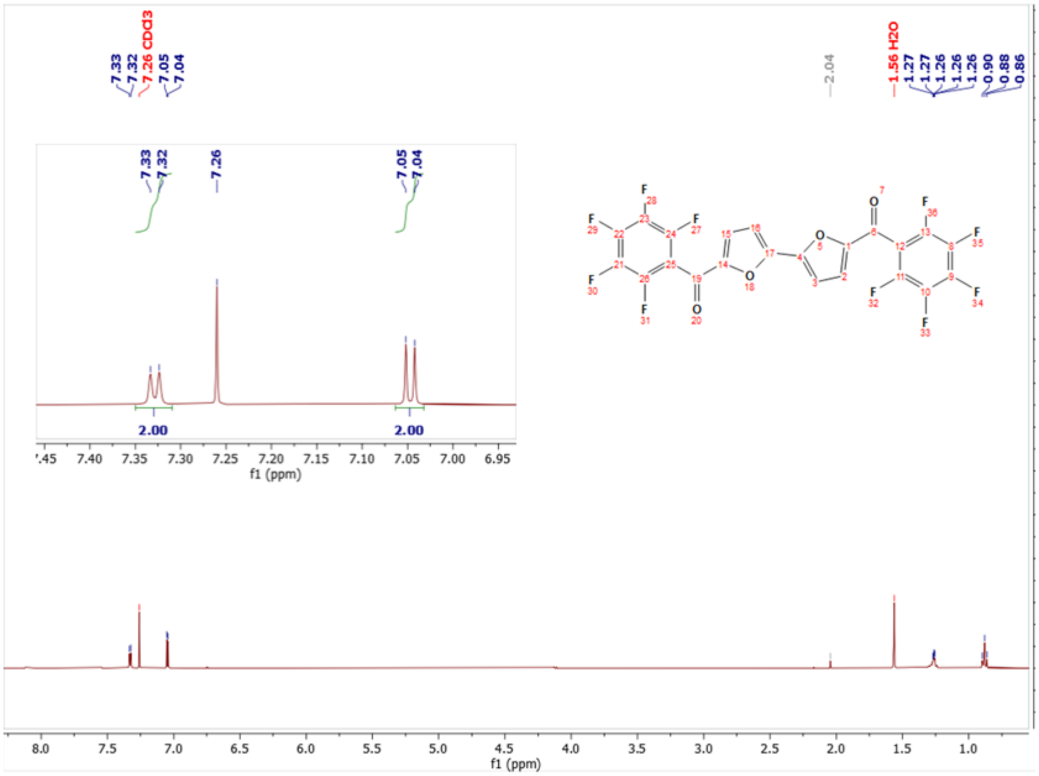

Figure S3. **¹H-NMR** spectrum of **F3** in chloroform-*d*

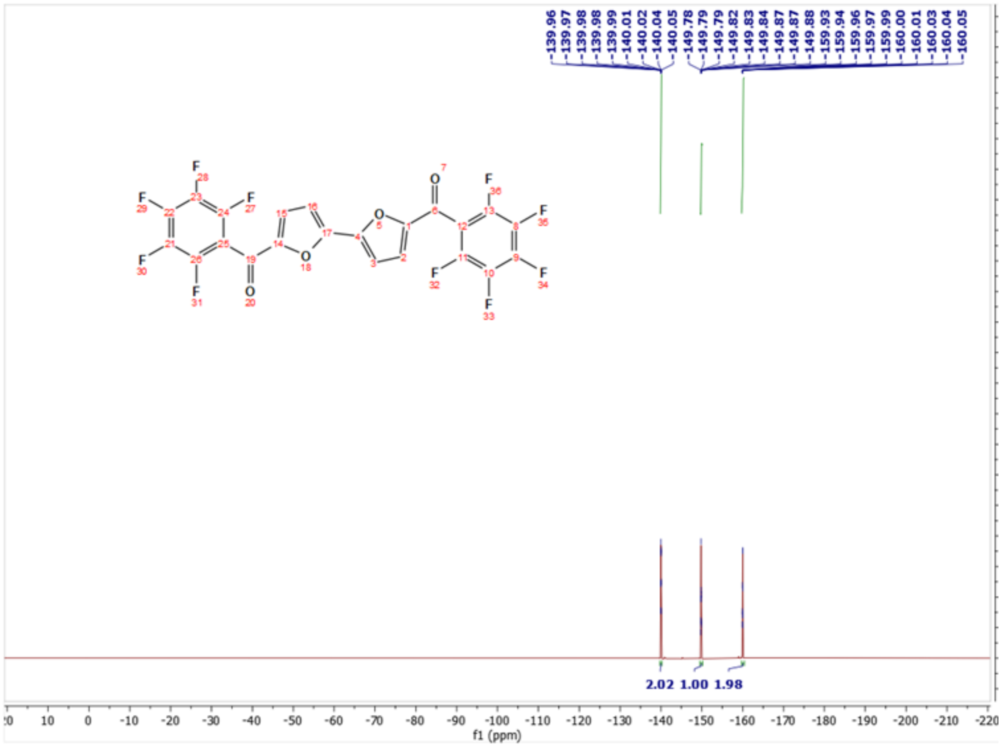

Figure S4. **¹⁹F-NMR** spectrum of **F3** in chloroform-*d*.

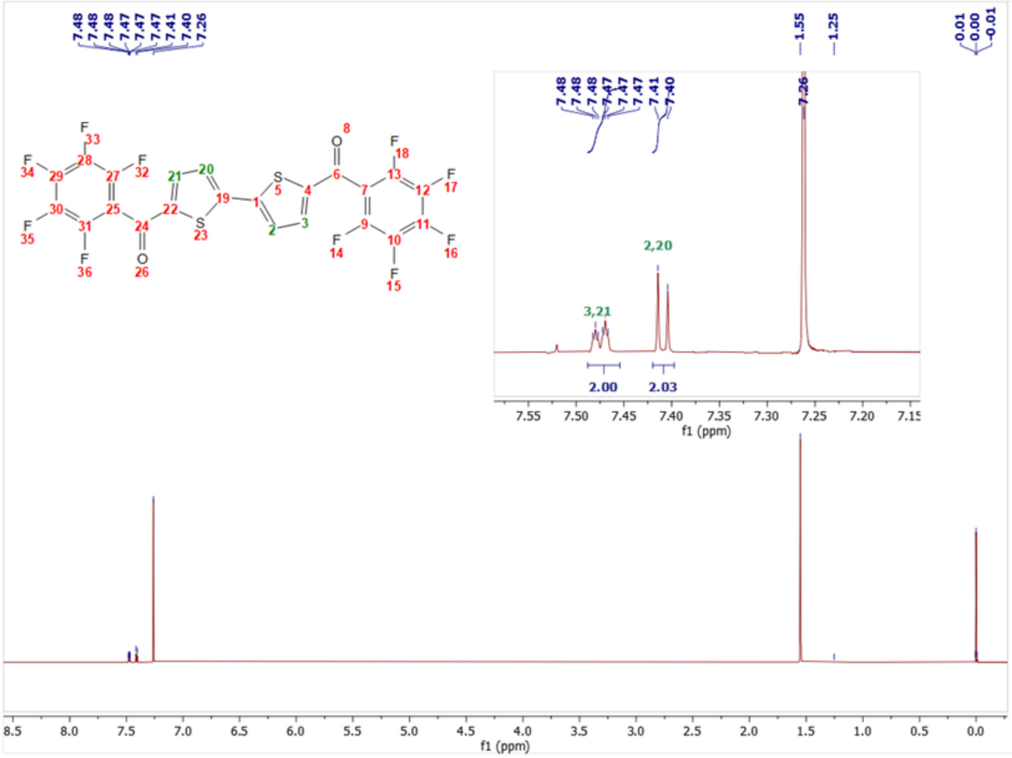

Figure S5. ^{1}**H-NMR** spectrum of **T3** in chloroform-*d*.

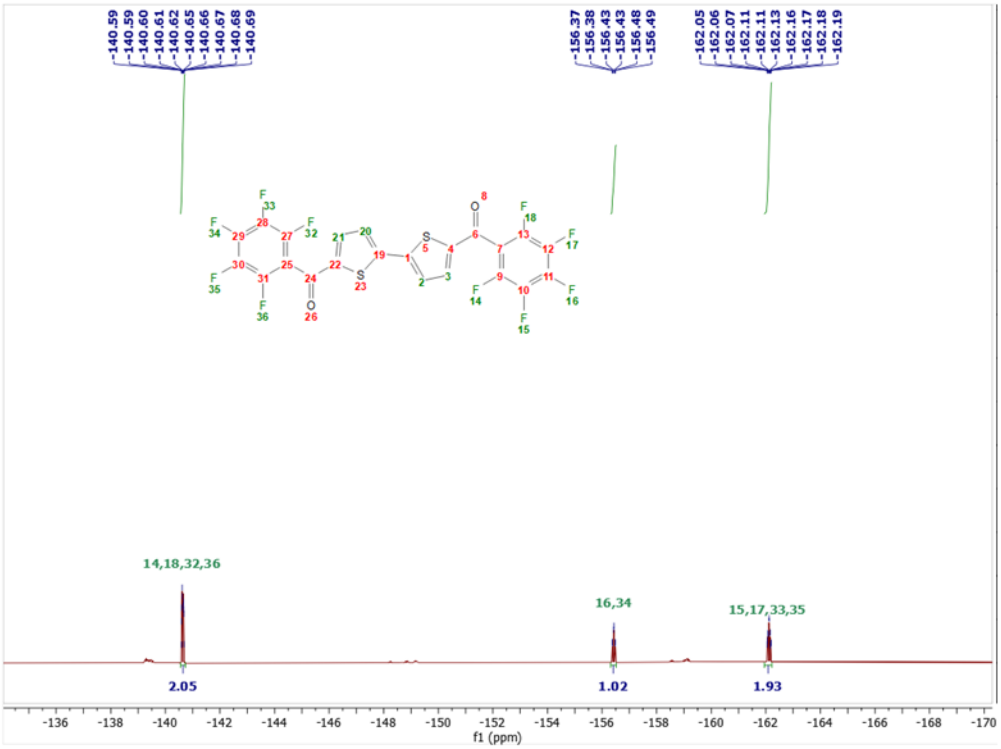

Figure S6. ^{19}**F-NMR** spectrum of **T3** in chloroform-*d*.

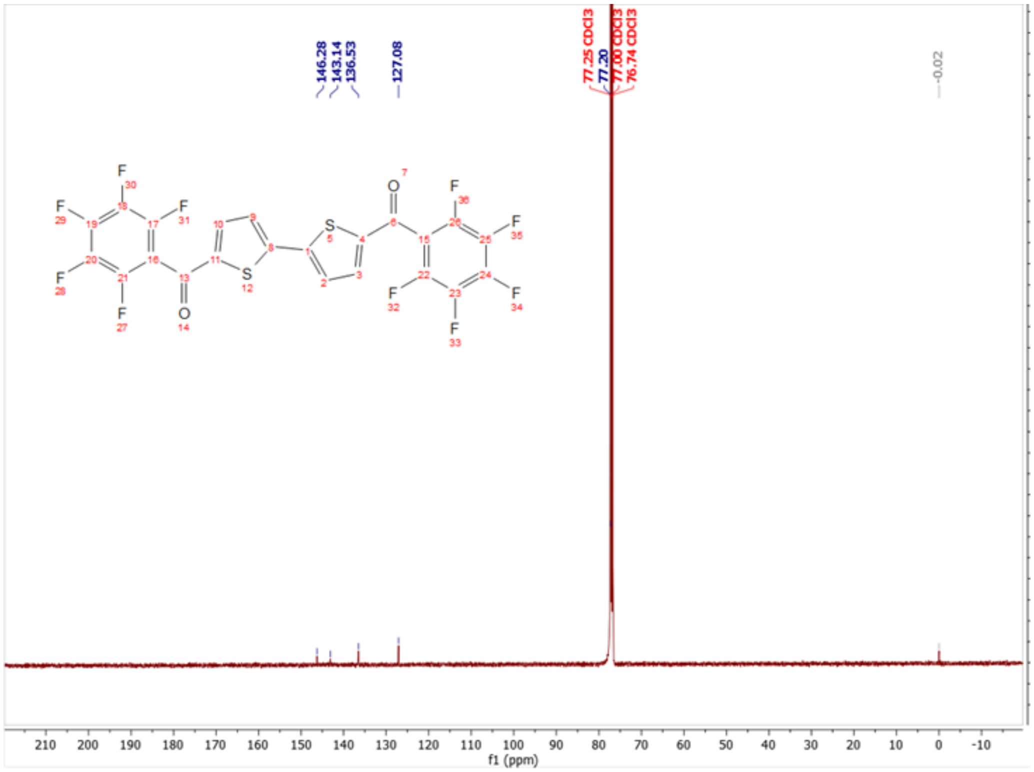

Figure S7. ^{13}**C-NMR** spectrum of **T3** in chloroform-*d*.

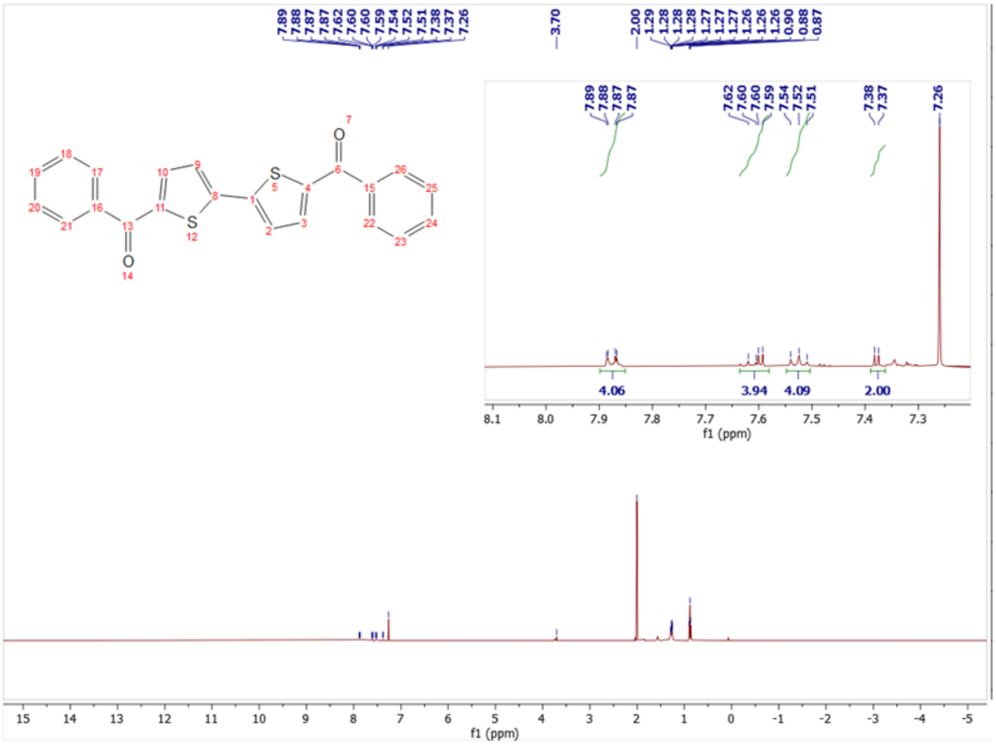

Figure S8. ^{1}**H-NMR** spectrum of **T2** in chloroform-*d*.

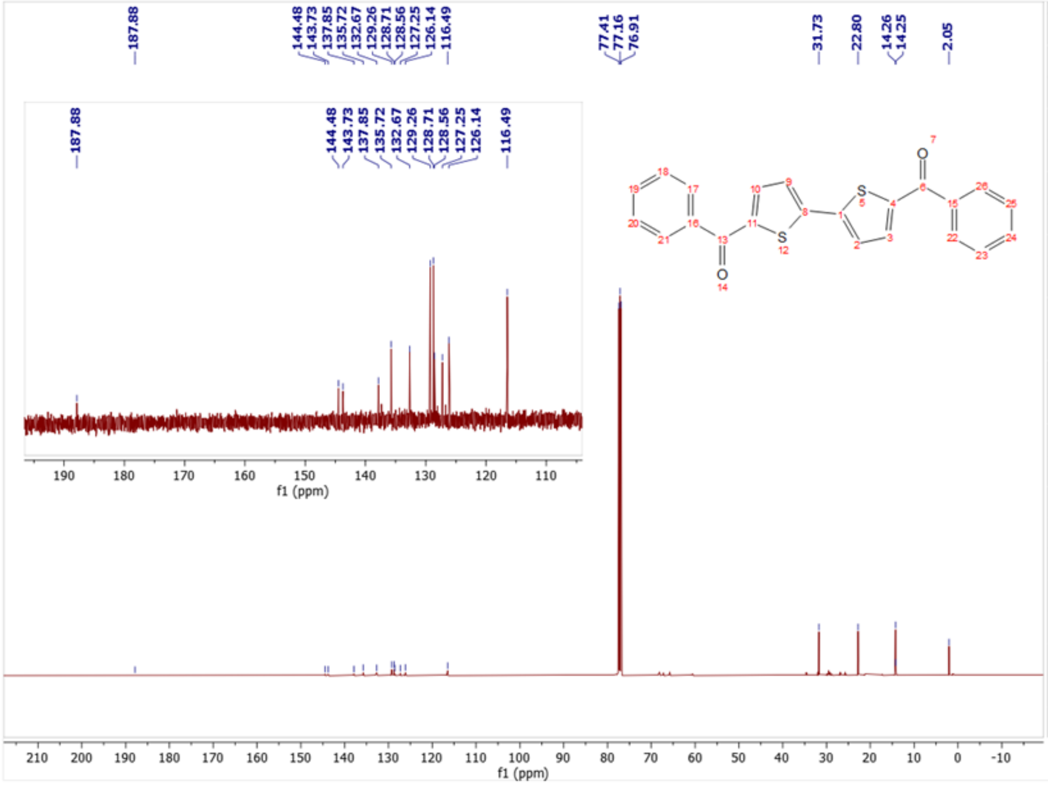

Figure S9. **^{13}C-NMR** spectrum of **T2** in chloroform-*d*.

S4. Calculations

	Absolute Energy (Hartree)
**F3**	-2139.882831
**F2**	-1147.63518
**F1**	-685.5125286
**T3**	-2785.840167
**T2**	-1793.592222
**T1**	-1331.4712899

Table S1. Calculated Absolute Energies of F1–F3 and T1–T3

Excitation energies and oscillator strengths:

F1

```
Excited State   1:      Singlet-A      3.3942 eV  365.28 nm  f=0.0000  <S**2>=0.000
      47 -> 51        0.29134
      48 -> 50        0.63566
```

F2

```
Excited State   1:      Singlet-A      3.3383 eV  371.40 nm  f=0.8445  <S**2>=0.000
      83 -> 90        0.15974
      84 -> 91        0.10804
      87 -> 90        0.18199
      89 -> 90        0.64529
```

F3

```
Excited State   1:      Singlet-A      3.3392 eV  371.30 nm  f=1.1158  <S**2>=0.000
     125 ->130       -0.16496
     129 ->130        0.67663
```

T1

```
Excited State   1:      Singlet-A      3.3500 eV  370.10 nm  f=0.0001  <S**2>=0.000
      55 -> 59        0.24730
      56 -> 58        0.65413
```

T2

```
Excited State   1:      Singlet-A      3.2061 eV  386.71 nm  f=0.9568  <S**2>=0.000
      90 -> 98       -0.11042
      97 -> 98        0.68839
```

T3

```
Excited State   1:      Singlet-A      3.1631 eV  391.97 nm  f=1.0521  <S**2>=0.000
     137 ->138        0.69574
```

Cartesian coordinates calculated at B3LYP/6-31G(d)

F1

Standard orientation:

Center Number	Atomic Number	Atomic Type	Coordinates (Angstroms) X	Y	Z
1	6	0	0.628238	-0.345998	-0.000013
2	6	0	0.953574	-1.686398	-0.000091
3	6	0	2.368249	-1.739841	0.000000
4	6	0	2.818232	-0.437767	0.000099
5	8	0	1.749982	0.418994	0.000099
6	1	0	0.253525	-2.509071	-0.000188
7	1	0	2.989783	-2.625056	-0.000013
8	6	0	-0.628239	0.345998	-0.000031
9	6	0	-0.953575	1.686398	-0.000110
10	8	0	-1.749983	-0.418994	0.000039
11	6	0	-2.368250	1.739841	-0.000065
12	1	0	-0.253526	2.509072	-0.000177
13	6	0	-2.818233	0.437767	0.000012
14	1	0	-2.989785	2.625056	-0.000093
15	6	0	4.164509	0.113800	0.000222
16	8	0	4.433952	1.301368	-0.000007
17	1	0	4.953779	-0.667989	0.000105
18	6	0	-4.164509	-0.113801	0.000086
19	8	0	-4.433947	-1.301370	-0.000171
20	1	0	-4.953778	0.667988	0.000033

F2

Standard orientation:

Center Number	Atomic Number	Atomic Type	Coordinates (Angstroms) X	Y	Z
1	6	0	-2.857678	-0.056472	-0.176309
2	6	0	-2.633782	1.303875	-0.204126
3	6	0	-1.229964	1.495665	-0.226283
4	6	0	-0.678204	0.233652	-0.210696
5	8	0	-1.652685	-0.710656	-0.188013
6	1	0	-3.392870	2.071483	-0.234953
7	1	0	-0.684622	2.427692	-0.256844
8	6	0	0.678222	-0.233780	-0.210658
9	6	0	1.230006	-1.495785	-0.226202
10	8	0	1.652689	0.710546	-0.187931
11	6	0	2.633817	-1.303980	-0.204046
12	1	0	0.684671	-2.427815	-0.256806
13	6	0	2.857671	0.056380	-0.176275
14	1	0	3.392898	-2.071591	-0.234906
15	6	0	4.054236	0.912965	-0.251985
16	8	0	3.949829	2.089905	-0.581692
17	6	0	-4.054275	-0.913035	-0.251942
18	8	0	-3.949941	-2.089991	-0.581604
19	6	0	5.392952	0.307664	0.041810
20	6	0	5.581577	-0.736779	0.959593
21	6	0	6.510210	0.869191	-0.596105
22	6	0	6.863777	-1.222510	1.218233
23	1	0	4.732877	-1.145795	1.498184
24	6	0	7.786487	0.373391	-0.347944
25	1	0	6.352683	1.694579	-1.282928
26	6	0	7.965724	-0.675629	0.559037
27	1	0	7.001968	-2.022689	1.940229
28	1	0	8.643877	0.805748	-0.856468
29	1	0	8.963063	-1.059088	0.757261
30	6	0	-5.392974	-0.307634	0.041826
31	6	0	-5.581538	0.736756	0.959685
32	6	0	-6.510254	-0.869022	-0.596162
33	6	0	-6.863711	1.222556	1.218334
34	1	0	-4.732809	1.145666	1.498315
35	6	0	-7.786502	-0.373125	-0.348022
36	1	0	-6.352778	-1.694369	-1.283044
37	6	0	-7.965681	0.675833	0.559043
38	1	0	-7.001863	2.022687	1.940392
39	1	0	-8.643918	-0.805356	-0.856610
40	1	0	-8.963000	1.059346	0.757263

F3

Standard orientation:

Center Number	Atomic Number	Atomic Type	Coordinates (Angstroms) X	Y	Z
1	6	0	-2.843878	-0.066337	0.224889
2	6	0	-2.704490	-0.071881	-1.147024
3	6	0	-1.317189	-0.075914	-1.420956
4	6	0	-0.690751	-0.071345	-0.191812
5	8	0	-1.604820	-0.067105	0.809597
6	1	0	-3.507591	-0.075808	-1.869470
7	6	0	0.690804	-0.071343	0.191849
8	6	0	1.317260	-0.075653	1.420984
9	8	0	1.604846	-0.067411	-0.809576
10	6	0	2.704557	-0.071748	1.147030
11	6	0	2.843948	-0.066655	-0.224879
12	1	0	3.507635	-0.075583	1.869505
13	6	0	3.989215	-0.096113	-1.129766
14	8	0	3.867249	-0.195240	-2.340083
15	6	0	-3.989215	-0.095701	1.129711
16	8	0	-3.867284	-0.194641	2.340051
17	6	0	5.367364	-0.038973	-0.503607
18	6	0	5.783463	1.014710	0.313331
19	6	0	6.317484	-1.010502	-0.837769
20	6	0	7.087123	1.102005	0.794422
21	6	0	7.623096	-0.949029	-0.359068
22	6	0	8.009065	0.114912	0.454478
23	6	0	-5.367309	-0.038724	0.503554
24	6	0	-5.783613	1.014715	-0.313580
25	6	0	-6.317337	-1.010286	0.838001
26	6	0	-7.087292	1.101766	-0.794663
27	6	0	-7.622971	-0.949058	0.359318
28	6	0	-8.009118	0.114677	-0.454430
29	9	0	-4.925743	1.991610	-0.649001
30	9	0	-7.459249	2.126184	-1.568691
31	9	0	-9.259242	0.183719	-0.912366
32	9	0	-8.506203	-1.901469	0.671975
33	9	0	-5.978096	-2.050896	1.603396
34	9	0	5.978345	-2.051328	-1.602977
35	9	0	8.506457	-1.901422	-0.671475
36	9	0	9.259160	0.184217	0.912457
37	9	0	7.458956	2.126623	1.568247
38	9	0	4.925459	1.991569	0.648427
39	1	0	0.828005	-0.081650	2.384032
40	1	0	-0.827889	-0.082192	-2.383980

T1

Standard orientation:

Center Number	Atomic Number	Atomic Type	Coordinates (Angstroms) X	Y	Z
1	6	0	0.681983	-0.242124	0.000135
2	6	0	1.120959	-1.557906	0.000344
3	6	0	2.526910	-1.672362	0.000337
4	6	0	3.170945	-0.450760	0.000111
5	1	0	0.447331	-2.408041	0.000546
6	1	0	3.059099	-2.618149	0.000510
7	6	0	-0.681983	0.242124	0.000107
8	6	0	-1.120959	1.557906	0.000322
9	6	0	-2.526910	1.672362	0.000249
10	1	0	-0.447332	2.408042	0.000574
11	6	0	-3.170945	0.450760	-0.000034
12	1	0	-3.059099	2.618149	0.000416
13	6	0	4.605236	-0.194964	0.000009
14	8	0	5.110368	0.914655	-0.000221
15	1	0	5.228007	-1.115072	-0.000154
16	6	0	-4.605236	0.194964	-0.000212
17	8	0	-5.110368	-0.914655	-0.000301
18	1	0	-5.228007	1.115072	-0.000202
19	16	0	-2.031850	-0.870809	-0.000237
20	16	0	2.031850	0.870809	-0.000121

T2

Standard orientation:

Center Number	Atomic Number	Atomic Type	X	Y	Z
1	6	0	-3.190511	-0.353679	-0.147333
2	6	0	-2.868460	0.978942	-0.323155
3	6	0	-1.477203	1.216639	-0.374837
4	6	0	-0.721176	0.063222	-0.242625
5	1	0	-3.612756	1.756395	-0.445068
6	1	0	-1.039943	2.198004	-0.525085
7	6	0	0.721228	-0.063003	-0.242651
8	6	0	1.477234	-1.216426	-0.374954
9	6	0	2.868498	-0.978762	-0.323240
10	1	0	1.039954	-2.197766	-0.525300
11	6	0	3.190584	0.353827	-0.147289
12	1	0	3.612782	-1.756224	-0.445191
13	6	0	4.487881	1.054359	-0.121314
14	8	0	4.524015	2.273100	-0.281743
15	6	0	-4.487789	-1.054274	-0.121339
16	8	0	-4.523828	-2.273013	-0.281758
17	6	0	5.753626	0.277874	0.080323
18	6	0	5.833208	-0.866768	0.888037
19	6	0	6.921024	0.775084	-0.520536
20	6	0	7.056361	-1.511860	1.074347
21	1	0	4.949196	-1.231768	1.400686
22	6	0	8.136333	0.119547	-0.347787
23	1	0	6.851980	1.677704	-1.119052
24	6	0	8.206198	-1.026949	0.449441
25	1	0	7.111366	-2.389061	1.713166
26	1	0	9.031790	0.503865	-0.828472
27	1	0	9.156584	-1.534894	0.589906
28	6	0	-5.753619	-0.277915	0.080297
29	6	0	-5.833346	0.866786	0.887920
30	6	0	-6.920975	-0.775363	-0.520446
31	6	0	-7.056590	1.511702	1.074230
32	1	0	-4.949375	1.231968	1.400500
33	6	0	-8.136378	-0.119997	-0.347707
34	1	0	-6.851819	-1.678029	-1.118880
35	6	0	-8.206381	1.026560	0.449419
36	1	0	-7.111706	2.388941	1.712988
37	1	0	-9.031801	-0.504486	-0.828317
38	1	0	-9.156843	1.534361	0.589888
39	16	0	-1.748568	-1.338905	-0.063693
40	16	0	1.748606	1.339093	-0.063591

T3

Standard orientation:

Center Number	Atomic Number	Atomic Type	Coordinates (Angstroms) X	Y	Z
1	6	0	-3.151890	0.574448	0.036496
2	6	0	-2.925290	-0.787141	0.112810
3	6	0	-1.556353	-1.120918	0.131498
4	6	0	-0.723354	-0.012906	0.068904
5	1	0	-3.721655	-1.520062	0.163204
6	6	0	0.723271	0.013454	0.068951
7	6	0	1.556305	1.121371	0.132757
8	6	0	2.925228	0.787557	0.113765
9	6	0	3.151731	-0.573966	0.035920
10	1	0	3.721621	1.520393	0.164935
11	6	0	4.400252	-1.333695	0.022782
12	8	0	4.414079	-2.556590	0.045293
13	6	0	-4.400482	1.334028	0.024191
14	8	0	-4.414517	2.556880	0.047954
15	6	0	5.708357	-0.570265	0.012433
16	6	0	6.051118	0.322444	-1.005171
17	6	0	6.673714	-0.839983	0.988472
18	6	0	7.299349	0.937646	-1.053216
19	6	0	7.923438	-0.227397	0.966350
20	6	0	8.237679	0.659288	-0.062230
21	6	0	-5.708461	0.570291	0.013045
22	6	0	-6.051122	-0.321080	-1.005766
23	6	0	-6.673738	0.838391	0.989607
24	6	0	-7.299208	-0.936515	-1.054494
25	6	0	-7.923322	0.225529	0.966805
26	6	0	-8.237464	-0.659780	-0.062981
27	9	0	-5.175705	-0.598086	-1.984771
28	9	0	-7.602953	-1.779553	-2.046295
29	9	0	-9.434234	-1.246341	-0.095427
30	9	0	-8.821159	0.478328	1.923287
31	9	0	-6.400402	1.671323	1.997541
32	9	0	6.400293	-1.674305	1.995229
33	9	0	8.821365	-0.481807	1.922326
34	9	0	9.434615	1.245523	-0.094015
35	9	0	7.603136	1.781943	-2.043931
36	9	0	5.175669	0.600999	-1.983717
37	1	0	1.187191	2.139024	0.200110
38	1	0	-1.187183	-2.138621	0.197756
39	16	0	-1.650491	1.465595	-0.012635
40	16	0	1.650451	-1.464975	-0.014214

Calculated Dimers

	F1		F2	F3
	1	2		
**HOMO**	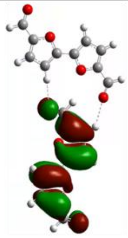	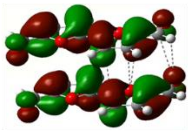	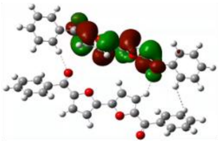	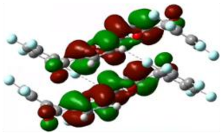
**LUMO**	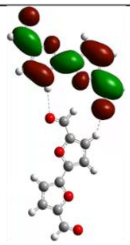	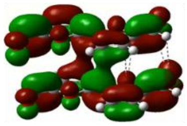	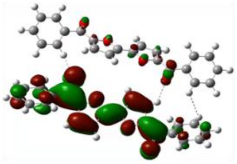	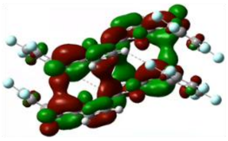

	T1		T2	T3
	1	2		
**HOMO**	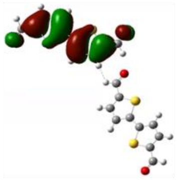	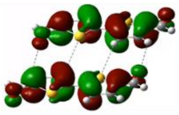	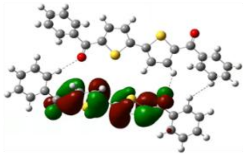	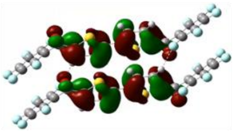
**LUMO**	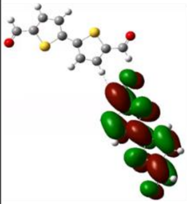	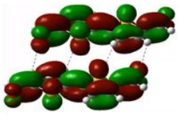	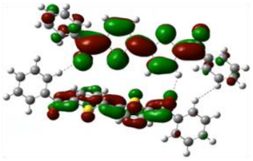	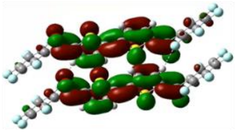

Figure S10. LUMO and HOMO surfaces of calculated dimer model based on the crystal data

	F1		F2	F3
	1	2		
HOMO-HOMO-1 [eV]	0.27	0.19	0.05	0.16
LUMO-LUMO+1 [eV]	0.27	0.08	0.08	0.27
	**T1**		**T2**	**T3**
	1	2		
HOMO-HOMO-1 [eV]	0.25	0.01	0.03	0.16
LUMO-LUMO+1 [eV]	0.27	0.19	0.05	0.002

Table S2. Calculated dimer orbital coupling

S5. X-ray structures of trimers

F3

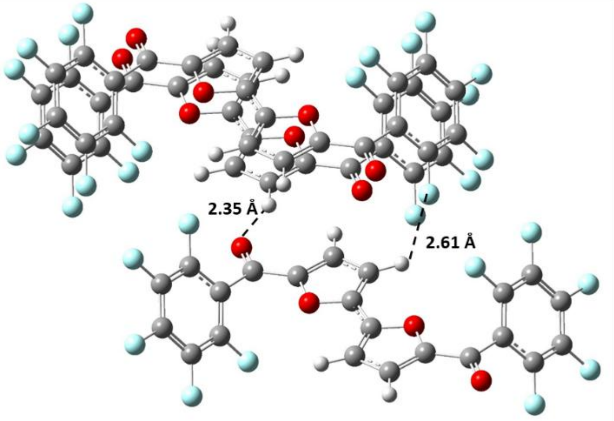

T3

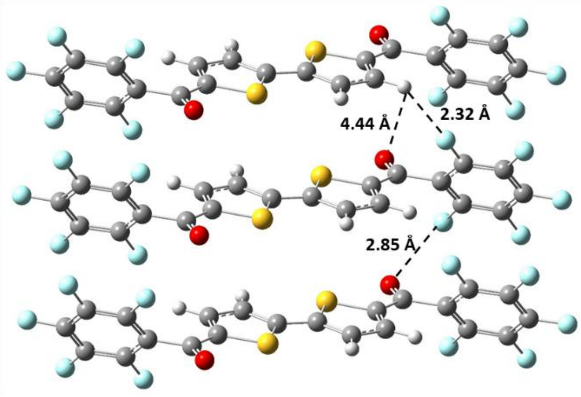

Figure S11. The X-ray structures of trimers of F3 (left) and T3 (right). For accuracy, the hydrogen atoms were optimized at the CAM-B3LYP/6-31-G(d)/D3 level.

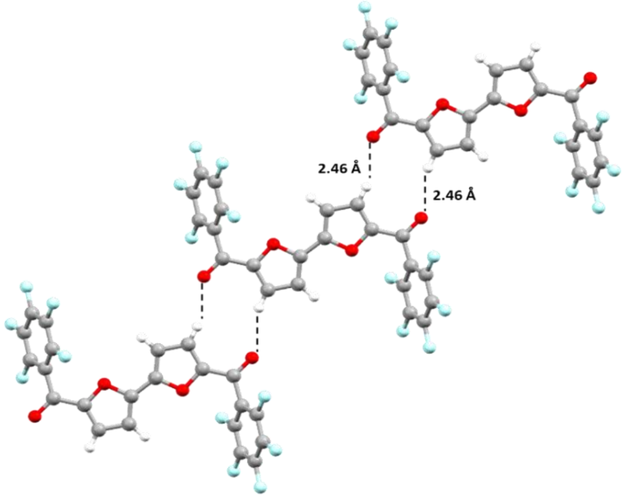

Figure S12. The X-ray structure of F3 showing C=O···H interactions

S6. Hirshfeld Surface Analysis

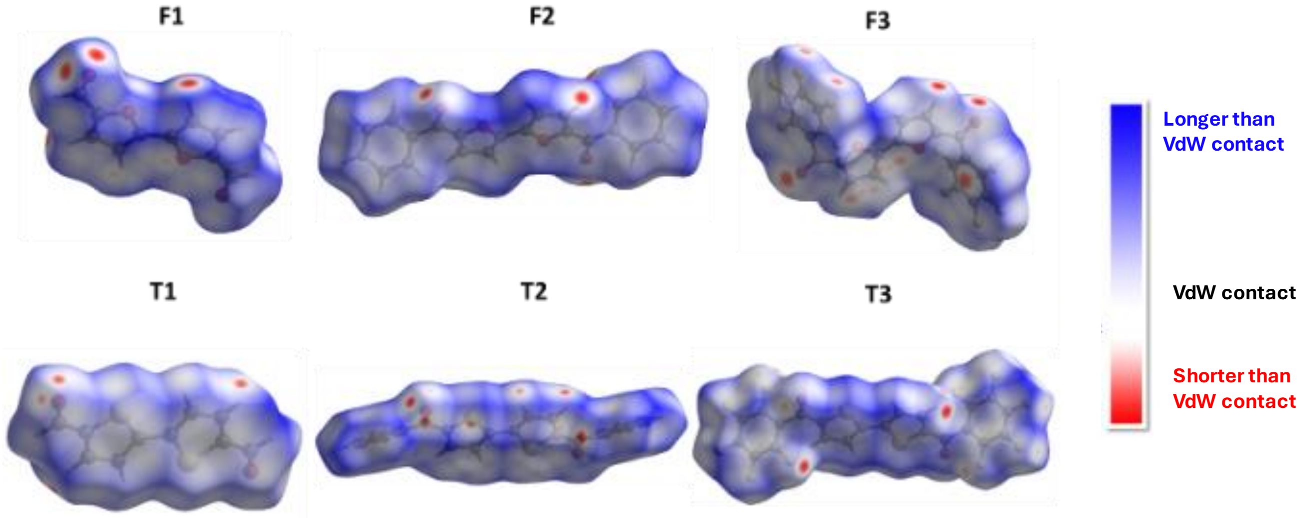

Figure S13. Hirshfeld Surface (HS) Analysis. Normalized sum of distance from HS to the nearest atom external to the surface and distance from HS to the nearest atom internal to the surface. Color coding was by CrystalExplorer. [11]

S7. Photophysical properties

	ε [$M^{-1}\cdot cm^{-1}$]	Solid		Solution		
		K_r	K_{nr}	K_r	K_{nr}	Stokes shifts [nm]
**F1**	28695	1.86×10^7	1.24×10^8	6.25×10^6	6.19×10^8	71
**F2**	37876	1.75×10^7	3.25×10^7	1.43×10^6	1.43×10^8	39
**F3**	23659	3.40×10^7	1.66×10^8	2.00×10^6	1.98×10^8	27
**T1**	21175	9.00×10^6	2.91×10^8	1.25×10^6	1.24×10^8	40
**T2**	34140	2.00×10^7	9.80×10^8	1.67×10^7	3.17×10^8	53
**T3**	33380	7.00×10^6	9.30×10^7	6.00×10^7	9.40×10^8	45

Table S3. Absorption coefficient (ε) measured in DCM, radiative (Kr) and non-radiative (Knr) rate constants [s^{-1}]

S8. Excitation spectra

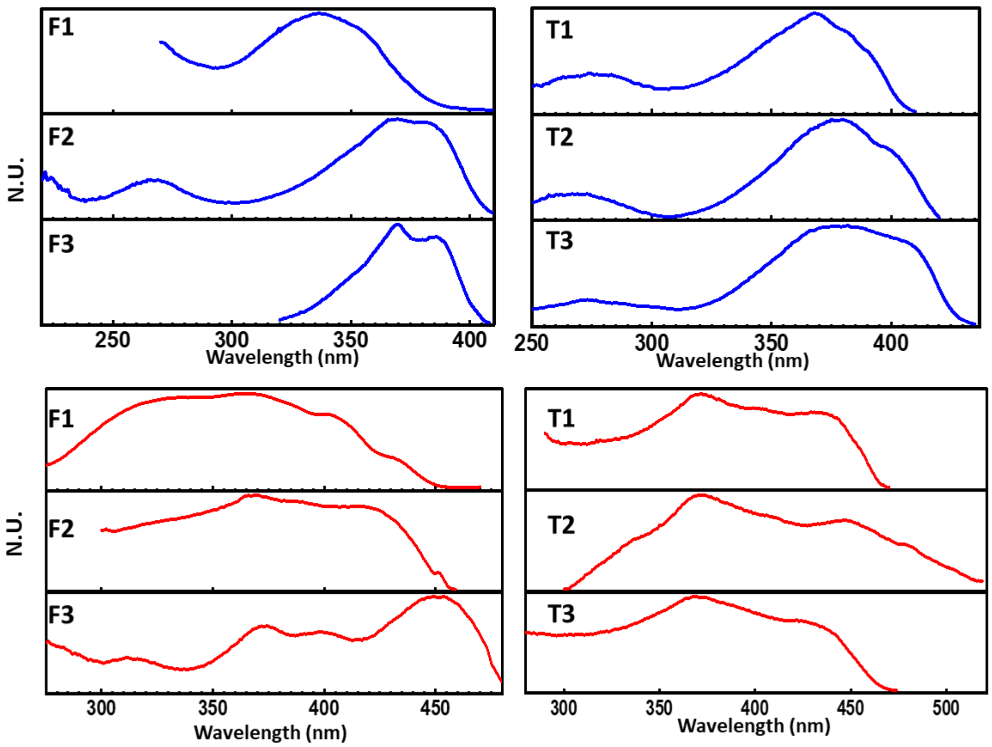

Figure S14. Normalized excitation spectra in DCM (blue) and solid (red)

S9. CIE 1931 coordinate diagram

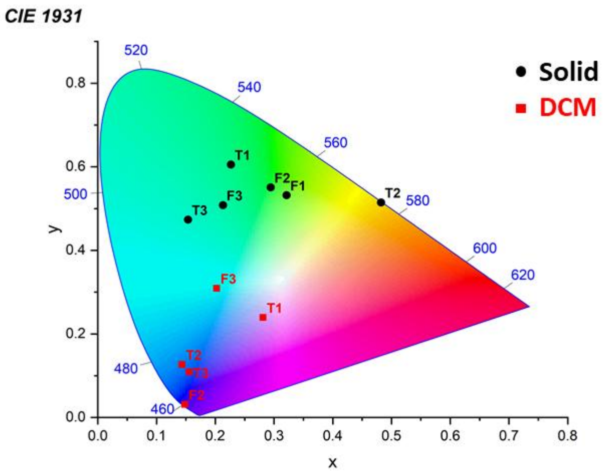

Figure S15. CIE 1931 coordinate diagram of compounds in solid state (black) and in DCM (red)

S10. Stability under ambient conditions

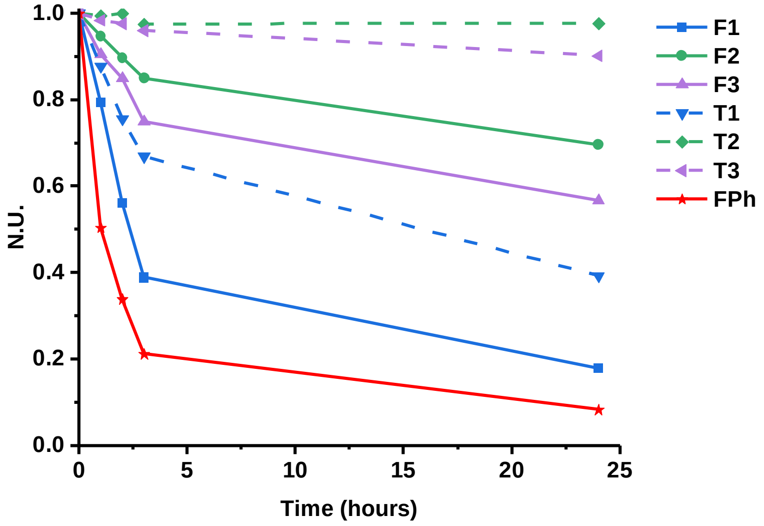

Figure S16. Decay in absorption spectra in 1,4-dioxane under ambient conditions of compounds F1-F3 and T1-T3 and compared with 5,5′-diphenyl-2,2′-bifuran (FPh)

S11. Photoluminescence at low temperature in solution and solid state

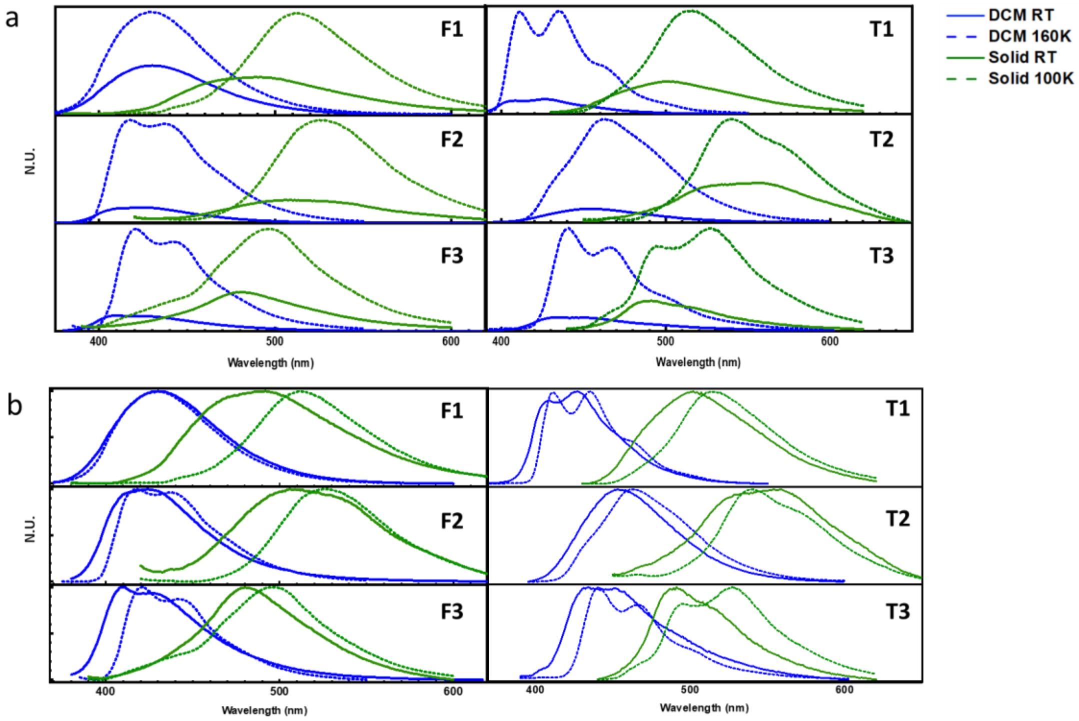

Figure S17. Emission spectra of F1–F3 and T1–T3 in dichloromethane (DCM; blue) and in the solid state (green) at room temperature (solid line) and low temperature (dashed line). (a) Normalized by the measurement at the lowest temperature and (b) per each measurement separately.

S12. PL in THF/water solutions

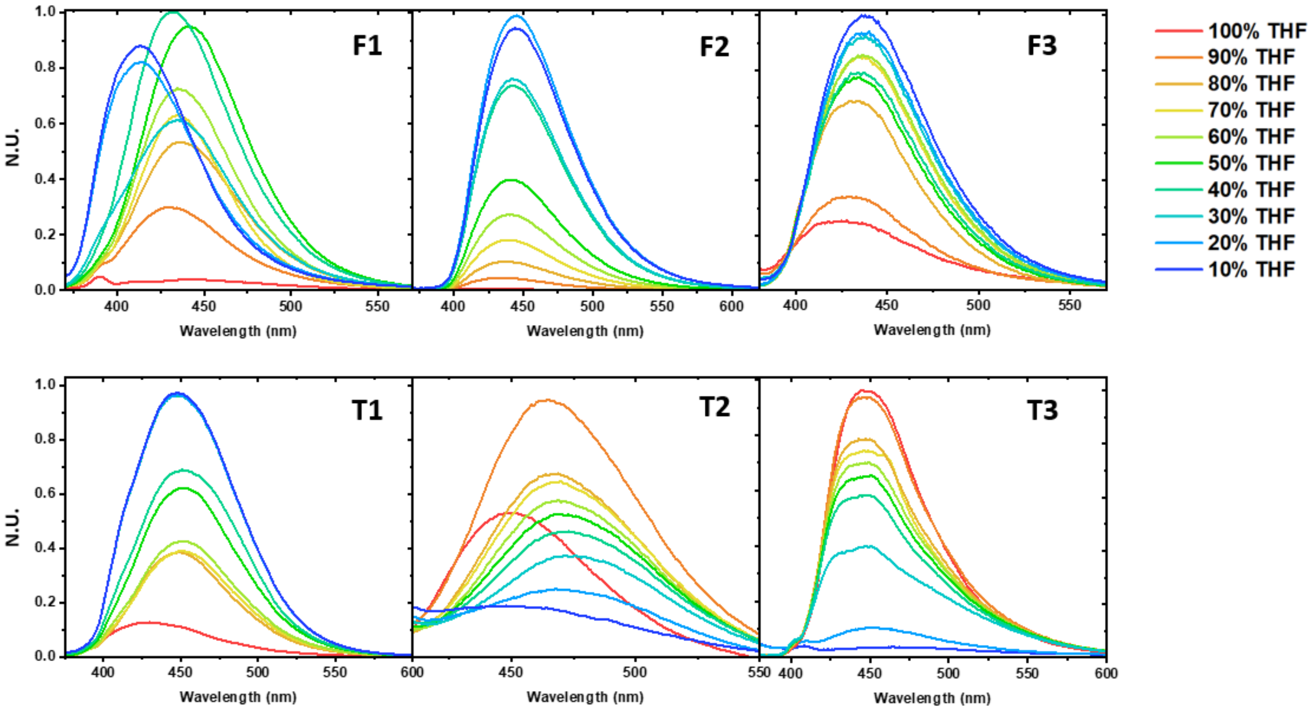

Figure S18. Fluorescence in THF/water mixtures. Excitation at 365nm, measured at room temperature.

S13. PL in PMMA

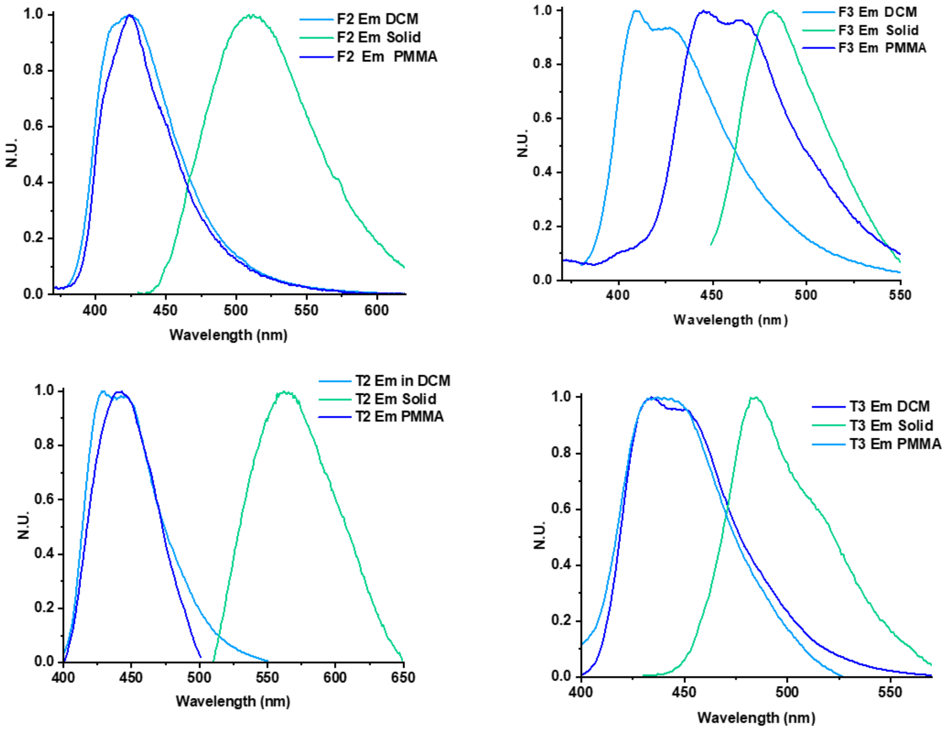

Figure S19. Comparison of emission spectra of F2–F3 and T2–T3 in DCM solution, as solids, and embedded in PMMA. Excitation wavelength for PMMA: F2 at 310nm, F3 at 350nm, T2 at 360nm and T3 at 370nm.

S14. Measurements in different solvents

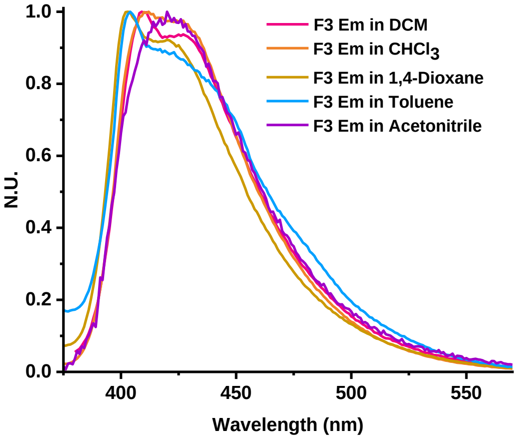

Figure S20. Comparison of emission spectra of F3 in different solvents

S15. Lifetime Measurements

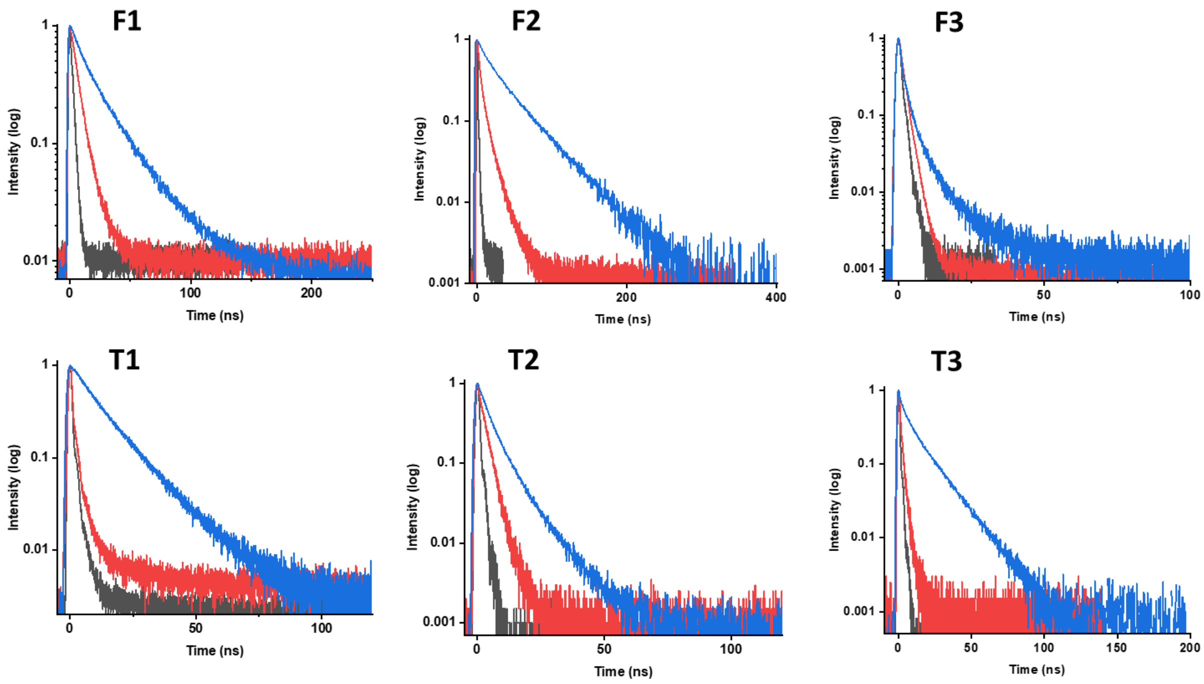

Figure S21. Lifetime Decay in DCM (black), solid at room temperature (red) and solid at 100K (blue)

	F1	F2	F3	T1	T2	T3
**Solution**	1.7573	1.4860	1.1752	1.2110	0.7711	1.2586
**Solid RT**	1.1698	1.7227	1.6747	1.8975	1.4539	1.5183
**Solid 100K**	1.0337	1.0419	1.3711	1.0654	1.6322	1.2138

Table S4. χ^2 for decay fits

S16. J-aggregation calculations

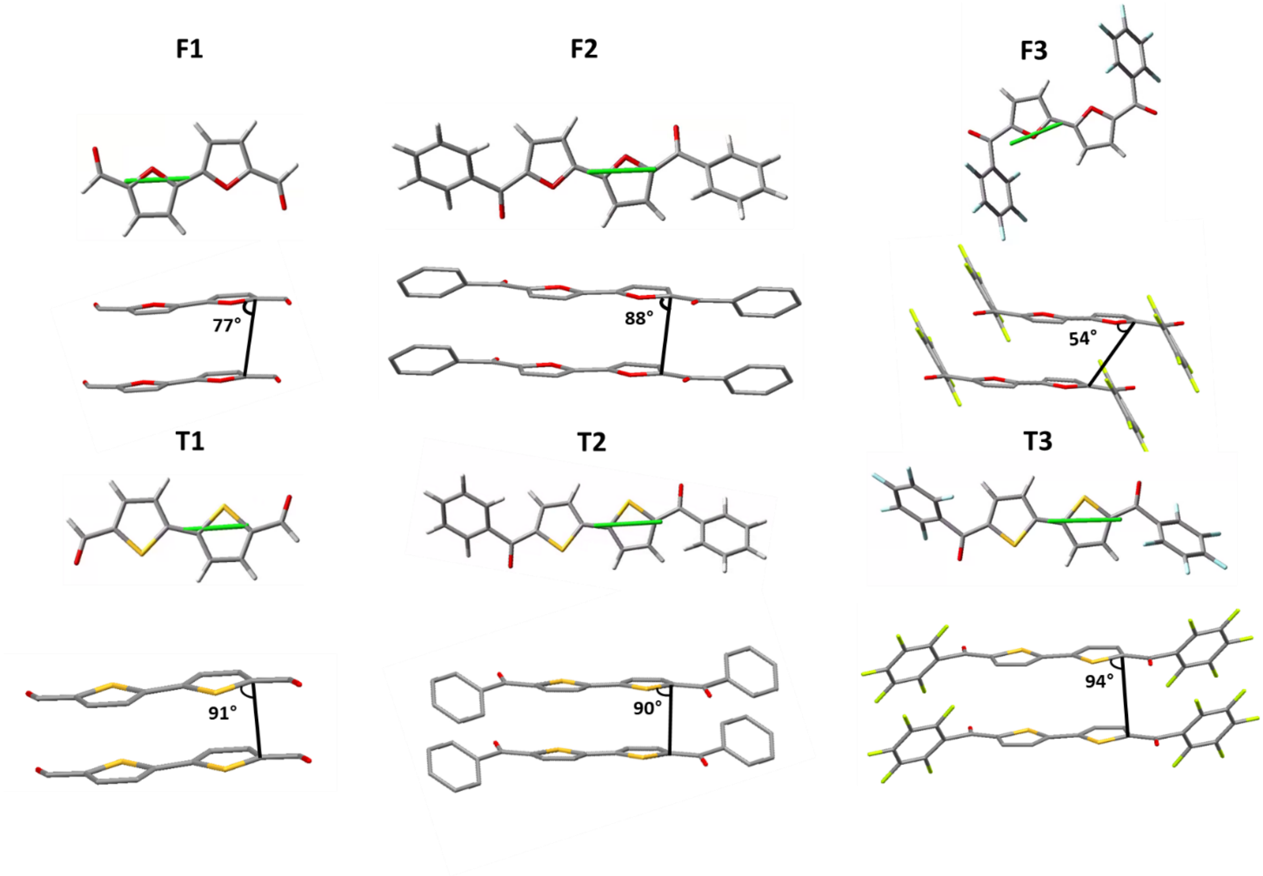

Figure S22. Transition dipole moment calculated using TDDFT with CAM-B3LYP/6-311G(d) (top) and the angle between the dipole moment and the neighboring molecule (bottom).

S17. X-Ray Data Collection and Structure Refinement

**Crystal data and structure refinement for F1**	
Empirical formula	$C_{10}H_6O_4$
Formula weight	190.15
Temperature/K	291.12(10)
Crystal system	monoclinic
Space group	$P2_1/n$
a/Å	3.7292(2)
b/Å	14.8371(11)
c/Å	7.5165(5)
α/°	90
β/°	96.755(7)
γ/°	90
Volume/Å^3	413.00(5)
Z	2
ρ_{calc}g/cm^3	1.529
μ/mm^{-1}	0.120
F(000)	196.0
Crystal size/mm^3	0.269 × 0.109 × 0.075
Radiation	Mo Kα (λ = 0.71073)
2Θ range for data collection/°	7.746 to 64.334
Index ranges	-5 ≤ h ≤ 4, -19 ≤ k ≤ 20, -10 ≤ l ≤ 11
Reflections collected	2880
Independent reflections	1167 [R_{int} = 0.0159, R_{sigma} = 0.0215]
Data/restraints/parameters	1167/0/64
Goodness-of-fit on F^2	1.052
Final R indexes [I>=2σ (I)]	R_1 = 0.0387, wR_2 = 0.1023
Final R indexes [all data]	R_1 = 0.0504, wR_2 = 0.1083
Largest diff. peak/hole / e Å^{-3}	0.20/-0.19
CCDC Deposition Number	2443169

Crystal data and structure refinement for F2	
Empirical formula	$C_{22}H_{14}O_4$
Formula weight	342.33
Temperature/K	293.7(1)
Crystal system	monoclinic
Space group	$P2_1/c$
a/Å	18.94271(174)
b/Å	3.8276(2)
c/Å	11.3496(7)
α/°	90
β/°	99.474(7)
γ/°	90
Volume/Å^3	811.65(9)
Z	2
ρ_{calc}g/cm^3	1.401
μ/mm^{-1}	0.097
F(000)	356.0
Crystal size/mm^3	0.136 × 0.115 × 0.03
Radiation	Mo Kα (λ = 0.71073)
2Θ range for data collection/°	6.542 to 64.23
Index ranges	-23 ≤ h ≤ 25, -4 ≤ k ≤ 5, -14 ≤ l ≤ 16
Reflections collected	8069
Independent reflections	2365 [R_{int} = 0.0503, R_{sigma} = 0.0591]
Data/restraints/parameters	2365/0/146
Goodness-of-fit on F^2	1.024
Final R indexes [I>=2σ (I)]	R_1 = 0.0535, wR_2 = 0.1174
Final R indexes [all data]	R_1 = 0.1069, wR_2 = 0.1365
Largest diff. peak/hole / e Å^{-3}	0.27/-0.17
CCDC Deposition Number	2418475

Crystal data and structure refinement for F3	
Empirical formula	$C_{11}H_2F_5O_2$
Formula weight	261.13
Temperature/K	150.1(3)
Crystal system	triclinic
Space group	P-1
a/Å	4.3097(2)
b/Å	7.7714(2)
c/Å	14.5880(7)
α/°	87.348(3)
β/°	82.055(4)
γ/°	77.873(3)
Volume/Å^3	473.02(3)
Z	2
ρ_{calc}g/cm^3	1.833
μ/mm^{-1}	0.190
F(000)	258.0
Crystal size/mm^3	0.3 × 0.192 × 0.127
Radiation	Mo Kα (λ = 0.71073)
2Θ range for data collection/°	5.362 to 64.154
Index ranges	-6 ≤ h ≤ 6, -11 ≤ k ≤ 9, -21 ≤ l ≤ 21
Reflections collected	8011
Independent reflections	2706 [R_{int} = 0.0457, R_{sigma} = 0.0493]
Data/restraints/parameters	2706/0/171
Goodness-of-fit on F^2	1.095
Final R indexes [I>=2σ (I)]	R_1 = 0.0470, wR_2 = 0.1210
Final R indexes [all data]	R_1 = 0.0601, wR_2 = 0.1304
Largest diff. peak/hole / e Å^{-3}	0.32/-0.24
CCDC Deposition Number	2418472

Crystal data and structure refinement for T2	
Empirical formula	$C_{22}H_{14}O_2S_2$
Formula weight	374.45
Temperature/K	210.0(1)
Crystal system	monoclinic
Space group	$P2_1/c$
a/Å	19.6082(7)
b/Å	3.8650(1)
c/Å	11.4067(4)
α/°	90
β/°	103.723(4)
γ/°	90
Volume/Å^3	839.79(5)
Z	2
ρ_{calc}g/cm^3	1.481
μ/mm^{-1}	0.331
F(000)	388.0
Crystal size/mm^3	0.27 × 0.07 × 0.03
Radiation	Mo Kα (λ = 0.71073)
2Θ range for data collection/°	4.276 to 64.228
Index ranges	-26 ≤ h ≤ 26, -5 ≤ k ≤ 5, -14 ≤ l ≤ 16
Reflections collected	13905
Independent reflections	2541 [R_{int} = 0.0274, R_{sigma} = 0.0233]
Data/restraints/parameters	2541/0/118
Goodness-of-fit on F^2	1.047
Final R indexes [I>=2σ (I)]	R_1 = 0.0315, wR_2 = 0.0839
Final R indexes [all data]	R_1 = 0.0406, wR_2 = 0.0887
Largest diff. peak/hole / e Å^{-3}	0.35/-0.19
CCDC Deposition Number	2418474

Crystal data and structure refinement for T3	
Empirical formula	$C_{22}H_4O_2F_{10}S_2$
Formula weight	554.37
Temperature/K	200.0(1)
Crystal system	triclinic
Space group	P-1
a/Å	4.1793(1)
b/Å	5.9316(2)
c/Å	19.6451(5)
α/°	86.130(2)
β/°	87.547(2)
γ/°	83.389(3)
Volume/Å^3	482.36(2)
Z	1
ρ_{calc}g/cm^3	1.908
μ/mm^{-1}	0.394
F(000)	274.0
Crystal size/mm^3	0.47 × 0.26 × 0.12
Radiation	Mo Kα (λ = 0.71073)
2Θ range for data collection/°	6.24 to 64.446
Index ranges	-5 ≤ h ≤ 5, -8 ≤ k ≤ 8, -28 ≤ l ≤ 29
Reflections collected	13055
Independent reflections	2957 [R_{int} = 0.0286, R_{sigma} = 0.0243]
Data/restraints/parameters	2957/0/163
Goodness-of-fit on F^2	1.065
Final R indexes [I>=2σ (I)]	R_1 = 0.0378, wR_2 = 0.0999
Final R indexes [all data]	R_1 = 0.0465, wR_2 = 0.1049
Largest diff. peak/hole / e Å^{-3}	0.39/-0.24
CCDC Deposition Number	2418473

S18. References

(1) Srinivasan, A.; Reddy, V. M.; Narayanan, S. J.; Sridevi, B.; Pushpan, S. K.; Ravikumar, M.; Chandrashekar, T. K. Tetrathia- and Tetraoxarubyrins: Aromatic, Core-Modified, Expanded Porphyrins. *Angew. Chem. Int. Ed. Engl.* **1997**, *36* (23), 2598–2601. https://doi.org/10.1002/anie.199725981.

(2) Itahara, T.; Hashimoto, M.; Yumisashi, H. Oxidative Dimerization of Thiophenes and Furans Bearing Electron-Withdrawing Substituents by Palladium Acetate. *Synthesis* **1984**, *1984* (03), 255–256. https://doi.org/10.1055/s-1984-30798.

(3) M. J. Frisch, G. W. Trucks, H. B. Schlegel, G. E. Scuseria, M. A. Robb, J. R. Cheeseman, et al. Gaussian 16 Rev. C.01, 2016.

(4) Calais, J.-L. Density-Functional Theory of Atoms and Molecules. R.G. Parr and W. Yang, Oxford University Press, New York, Oxford, 1989. IX + 333 Pp. Price £45.00. *Int. J. Quantum Chem.* **1993**, *47* (1), 101–101. https://doi.org/10.1002/qua.560470107.

(5) Lee, C.; Yang, W.; Parr, R. G. Development of the Colle-Salvetti Correlation-Energy Formula into a Functional of the Electron Density. *Phys. Rev. B Condens. Matter 37 2*, 785–789.

(6) Becke, A. D. Density-functional Thermochemistry. III. The Role of Exact Exchange. *J. Chem. Phys.* **1993**, *98* (7), 5648–5652. https://doi.org/10.1063/1.464913.

(7) Koch, W.; Holthausen, M. C. A Chemist’s Guide to Density Functional Theory; 2000.

(8) Dolomanov, O. V.; Bourhis, L. J.; Gildea, R. J.; Howard, J. A. K.; Puschmann, H. *OLEX2* : A Complete Structure Solution, Refinement and Analysis Program. *J. Appl. Crystallogr.* **2009**, *42* (2), 339–341. https://doi.org/10.1107/S0021889808042726.

(9) Sheldrick, G. M. *SHELXT* – Integrated Space-Group and Crystal-Structure Determination. *Acta Crystallogr. Sect. Found. Adv.* **2015**, *71* (1), 3–8. https://doi.org/10.1107/S2053273314026370.

(10) Sheldrick, G. M. Crystal Structure Refinement with *SHELXL*. *Acta Crystallogr. Sect. C Struct. Chem.* **2015**, *71* (1), 3–8. https://doi.org/10.1107/S2053229614024218.

(11) *CrystalExplorer*. https://crystalexplorer.net/docs/manual/isosurfaces/properties.
